# Polysialosides Outperform Sulfated Analogs for Binding with SARS‐CoV‐2

**DOI:** 10.1002/smll.202500719

**Published:** 2025-07-16

**Authors:** Vinod Khatri, Nico Boback, Hassan Abdelwahab, Daniela Niemeyer, Tahlia M. Palmer, Anil Kumar Sahoo, Yannic Kerkhoff, Kai Ludwig, Julian Heinze, Dilara Balci, Jakob Trimpert, Rainer Haag, Tatyana L. Povolotsky, Roland R. Netz, Christian Drosten, Daniel C. Lauster, Sumati Bhatia

**Affiliations:** ^1^ Freie Universität Berlin Institute of Chemistry and Biochemistry Takustr. 3 14195 Berlin Germany; ^2^ Department of Chemistry TDL Govt. College for Women Murthal Sonipat Haryana 131027 India; ^3^ Department of Chemistry, Faculty of Science and Engineering Swansea University Singleton Campus Swansea SA2 8PP United Kingdom; ^4^ Freie Universität Berlin Institute of Pharmacy, Biopharmaceuticals Kelchstr. 31 12169 Berlin Germany; ^5^ Institute of Virology, Campus Berlin Mitte Charité – Universitätsmedizin Berlin Charitéplatz 1 10117 Berlin Germany; ^6^ Freie Universität Berlin Department of Physics Arnimallee 14 14195 Berlin Germany; ^7^ Freie Universität Berlin Forschungszentrum für Elektronenmikroskopie, Core‐Facility BioSupraMol, Institute of Chemistry and Biochemistry Fabeckstraße 36a 14195 Berlin Germany; ^8^ IT & Data Services Zuse Institute Berlin Takustraße 7 14195 Berlin Germany; ^9^ Department of Pathobiology and Diagnostic Medicine Kansas State University 1800 Denison Avenue Manhattan KS 66506 USA; ^10^ Freie Universität Berlin SupraFAB, Department of Biology, Chemistry and Pharmacy, and Department of Physics Altensteinstr. 23a 14195 Berlin Germany

**Keywords:** MD simulations, polysialosides, SARS‐CoV‐2, virus binding

## Abstract

Both polysialosides and polysulfates are known to interact with the receptor binding domain (RBD) of the SARS‐CoV‐2 spike protein. However, a comprehensive site by site analysis of their binding affinities and potential synergistic antiviral effects have not been performed. Here, we report on the synthesis of polysialosides with nanomolar binding affinities to spike proteins of SARS‐CoV‐2 in solution using microscale thermophoresis. The dendritic polyglycerol based polysialosides dPG_500_SA_0.55_ and dPG_500_SA_0.25_, with a dissociation constant *K*_d_ of 4.78 nm and 10.85 nm, respectively, bind ≈500 times stronger than the high density polysulfated analog dPG_500_S_0.55_, to intact SARS‐CoV‐2 virus particles or isolated spike protein. In fact, the presence of sulfate groups in a heteromultivalent compound dPG_500_SA_0.20_S_0.20_ weakens the binding to spike proteins. A polycarboxylated analog does not bind to SARS‐CoV‐2, ruling out that the interaction of polysialoside is simply driven by electrostatics. Using explicit‐solvent all‐atom molecular dynamics simulations and ensemble docking studies, atomistic details are obtained on the interaction of different functional groups with the SARS‐CoV‐2 RBD. The data support the conclusion that sialosides interact stronger than sulfates for their binding with RBD of SARS‐CoV‐2. Notably, the most affine binder dPG_500_SA_0.55_ inhibits SARS‐CoV‐2 (WT, D614G) replication up to 98.6% at 0.5 µm concentrations.

## Graphical abstract

Both sulfated and sialylated glycans present on the cell surface act as co‐receptors for the SARS‐CoV‐2 attachment. Polysialylated and sulfated dendritic polyglycerols are studied for their binding with SARS‐CoV‐2. In solution, dendritic polyglycerol sialosides (dPGSAs) bind significantly stronger (low nm) to SARS‐CoV‐2 than their sulfated analogs. dPGSAs effectively inhibit the viral entry into Calu‐3 cells.

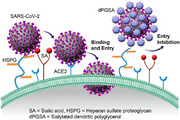

## 1 Introduction

The coronavirus disease COVID‐19, caused by the severe acute respiratory syndrome coronavirus 2 (SARS‐CoV‐2), which was first reported in Wuhan (China) in 2019, led to an acute global pandemic, with more than 776 million confirmed cases and over 7.0 million deaths (as of September 2024).^[^ ^1^ ^]^ Since the rise of SARS‐CoV‐2 there has been a great interest in understanding SARS‐CoV‐2 virus attachment and entry into host cells. SARS‐CoV‐2 is roughly globular with a diameter in the range of 80–120 nm, resulting in a surface area of 20–45 µm^2^.^[^ ^2^ ^]^ The viral membrane contains viral spike (S)‐glycoproteins, which are homotrimers consisting of S1 and S2 subunits. The S1 subunit of the (S)‐protein carries the receptor binding domains (RBD) that binds to the human angiotensin converting enzyme 2 (hACE2) on the surface of hosts cells, which mediates viral uptake.^[^ ^3^ ^]^

Many viruses exploit sialylated or sulfated glycans on cell membranes as a primary attachment factor before binding to specific membrane protein receptors needed for cell entry. During evolution of SARS‐CoV‐2 especially the role of polysulfates, such as glycosaminoglycans (GAG) became more important for viral attachment. This can be observed by an increased abundance of cationic amino acids on the RBD.^[^ ^4^, ^5^ ^]^ The RBD has a binding site for heparan sulfate lateral to the ACE2 binding site.^[^ ^6^ ^]^ Blocking of one or the other site of RBD with decoy structures has been demonstrated to be effective for virus inhibition.^[^ ^6^, ^7^, ^8^ ^]^ Interestingly, besides the relevance of sulfates, sialylated glycans were found to act as co‐receptors for the virus attachment. Saso and coworkers reported on the reduction of infection by SARS‐CoV‐2 after enzymatic removal of cell surface sialic acids or using lipidated 2,6‐sialyllactose linked to polyglutamic acid as a competitor for SARS‐CoV‐2 attachment to the host cell.^[^ ^9^ ^]^ Further, Nguyen et al. screened defined glycan libraries for binding with SARS‐CoV‐2 RBD and spike proteins using a catch and release ESI‐MS technique. They observed micromolar affinities of SARS‐CoV‐2 RBD interacting with sialylated glycolipids, thereby facilitating viral entry.^[^ ^10^ ^]^ Baker and coworkers even achieved apparent K_d_ values of 1 nm using surface plasmon resonance studies when highly sialylated glyconanoparticles were titrated against SARS‐CoV‐2.^[^ ^11^ ^]^ Another group with Petitjean observed a significant decrease in the infection of A549 cells by SARS‐CoV‐2 pseudoviruses at 10 µM using porphyrin‐based 9‐*O*‐acetyl sialoside oligomers.^[^ ^12^ ^]^ These observations on the interaction of SARS‐CoV‐2 RBD with sialosides and sulfates inspired us to explore different variants of polysialosides, polysulfates or hybrid materials displaying both functional entities. Using such defined nanostructures would not only have implications on virus inhibition, but also on the virus binding capability and preference towards sialosides or sulfates. As the receptor binding site for a sialoside has not been identified yet, one could identify from binding studies whether the binding sites overlap or are spatially separated. Thus, both functional groups could compete with each other or act synergistically.

Therefore, the study in hand investigates their roles in SARS‐CoV‐2 binding using dendritic polyglycerol (dPG) as carrier systems with similar sizes, geometry, and varying ligand densities (high and low). Also, both sialic acid and sulfate covalently linked to the same polymer have been explored. These polymer nanoparticles were then analyzed biophysically by means of Microscale thermophoresis (MST) with regard to their binding affinities towards different domains, namely the RBD or S1 subunit of SARS‐CoV‐2 S‐protein. For this purpose, multivalent sulfated and sialylated dendritic polyglycerols [dPGS and dPGSA], as well as heteromultivalent dPGs presenting both sialosides and sulfates [dPGSAS] were tested to bind to SARS‐CoV‐2 (WT, D614G). To investigate whether the aromatic group at the anomeric position of sialic acid contributes to its interaction with RBD, a polyglycerol‐based multivalent nanoparticle bearing aromatically modified sialosides [dPG(SA_aryl_)] was synthesized. Because each sialoside has one carboxylic acid group, a carboxylated PG analog [dPGC] was also explored to examine the role of multivalent carboxyl groups for the SA‐RBD interactions and to identify the importance of the electrostatic interaction. This was followed by MD simulations and ensemble docking studies to not only rationalize these binding behaviors but also to understand competition mechanisms of carboxylates, sulfates, and sialosides for their binding to SARS‐CoV‐2 RBD. We then conducted affinity measurements using MST of polymer nanoparticles against wild‐type SARS‐CoV‐2. Those nanoparticles with a detectable dissociation constant (K_d_) were further studied for their antiviral efficacy using entry inhibition assays on Calu‐3 cells. Virus titers were assessed 24 and 48 h post infection (hpi) using qPCR. In the presence of the highly sialylated polyglycerol dPG_500_SA_0.55_, SARS‐CoV‐2 infection was inhibited up to 98.6%. The interaction of polyglycerol sialosides with SARS‐CoV‐2 particles was further investigated and visualized by cryo‐electron tomography (cryo‐ET). Overall, our study identified highly sialylated polyglycerols as potential antivirals for inhibition of infection at early as well as later stages of SARS‐CoV‐2 infection. Additionally, our multivalent nanoparticles also provide evidence for the competition among sulfate and sialoside when they are presented together on the same dendritic polyglycerol for interactions with SARS‐CoV‐2 spike proteins.

## 2 Results and Discussion

### 2.1 Design, Synthesis and Characterization of Polyglycerol‐Based Nanoparticles

**Figure 1:**
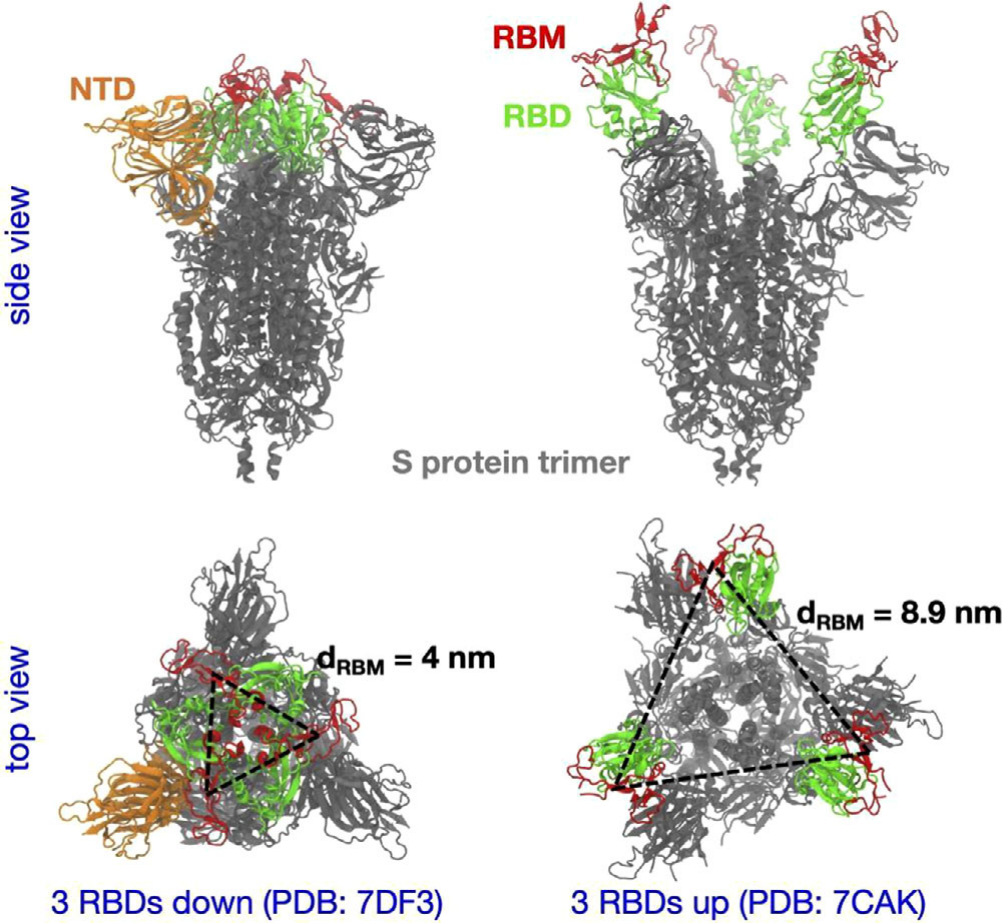
Deposited cryo‐electron microscopy structures (see PDB entries) of the spike (S) protein trimer with all three RBDs in the down or up conformation shown in the left and right column, respectively. The S‐protein is shown in grey, and the RBD in green except its receptor binding motif (RBM) that forms direct contacts with ACE2 is highlighted in red. The N‐terminal domain (NTD) of one monomer of the S‐protein is shown in orange only in the left column. The center‐of‐mass distance between two RBMs, dRBM, is mentioned for each conformation.

The SARS‐CoV‐2 spike (S)‐protein is a homotrimeric membrane protein with a globular head domain, being S1, and the stem region S2, which is required for fusion with the host cell membrane. The head domain S1 can be further divided into the N‐terminal domain (NTD) and the RBD, which interacts with ACE2 and attachment factors such as sulfates or sialosides. The RBD interacts with ACE2 via the receptor binding motif (RBM, see also **Figure** **1**). From the crystal structure of complete S‐proteins an intra‐trimeric distance between the center of RBDs of 4 and 8.9 nm could be determined when the RBD on the trimer is in down (PDB 7DF3) or up‐right (PDB 7CAK) conformation, respectively (Figure 1). In order to enhance the functional valency, i.e. the ability to bridge multiple receptor‐binding domains (RBDs) in either the upright or down conformation, we selected a 500 kDa dendritic polyglycerol (dPG_500_) with a hydrodynamic diameter (D_h_) of 13.21 nm. The high density of surface hydroxy groups, ≈21 ‐OH groups per nm^2^, allows further functionalization. The dPG_500_ was sialylated in three steps, according to a previously reported procedure, using a copper‐catalyzed Sharpless‐Huisgen click reaction^[^ ^13^ ^]^ to yield dPG_500_SA_0.25_ and dPG_500_SA_0.55_. For comparison, polysulfated analogs dPG_500_S_0.25_ and dPG_500_S_0.55_ were obtained with similar densities of functionalities according to a known sulfation protocol (see Supporting Information). Polysulfates were reported to bind with the lateral cationic patch on the RBD of SARS‐CoV‐2 spike proteins.^[^ ^8^ ^]^ Also, a recent study showed that self‐assembled polycarboxylated double layered sheets (up to >400 nm) could interact with S‐proteins via electrostatics.^[^ ^14^ ^]^ Therefore, to investigate the role of carboxylic acid groups of sialic acids in dPGSAs for SARS‐CoV‐2 binding, a polycarboxylated analog dPG_500_C_0.20_ with similar size and ζ‐potential as dPG_500_SA_0.25_ was synthesized, serving as control. Also in our previous work, we found that heteromultivalent polyglycoside systems targeting both hemagglutinin (HA) and neuraminidase (NA) of influenza A virus (IAV) outperformed homomultivalent compounds that target only HA or NA.^[^ ^15^ ^]^ Inspired by this, we aimed to translate the concept of heteromultivalency to SARS‐CoV‐2. In this case, however, we focused on targeting a single viral protein using two distinct ligand types. To this end, we synthesized the nanosystem dPG_500_SA_0.20_S_0.20,_ which presents both sialoside and sulfate groups on a single dendritic polyglycerol (dPG) scaffold.

All polymer conjugates based on dPG_100_ or dPG_500_ showed hydrodynamic diameters between 10 – 14 nm. Important to note is that the polysulfated and polysialylated analogs were similar in size, ligand density, and ζ‐potentials. For dPG_500_S_0.25_ and dPG_500_SA_0.25_ hydrodynamic diameters of D_h_ 13.3 and 14.6 nm, and ζ‐potentials of ‐26.2 and ‐28.2 mV respectively, were determined. For dPG_500_S_0.55_ and dPG_500_SA_0.55_ diameters of D_h_ 11.8 and 13.9 nm, and ζ‐potentials ‐36.3 and ‐45.9 mV respectively, were assessed.

**Scheme 1:**
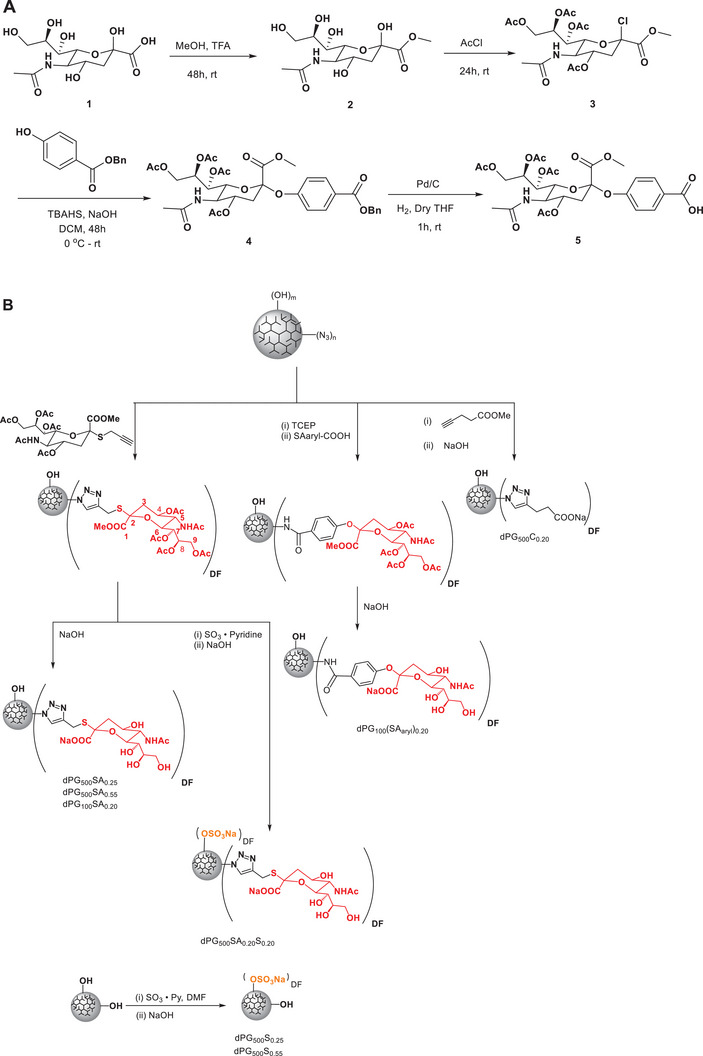
A) Synthesis of aromatically modified sialic acid; B) Overview of the synthesized multivalent nanoparticles carrying either sialosides, sulfates, carboxylates, or combinations thereof. Dendritic polyglycerols were used as scaffolds and modified via click‐chemistry or other conjugation strategies (see Supporting Information for synthesis details).

In a related context, sialic acids bearing aromatic substituents at the C‐2 position have been reported to inhibit influenza virus‐induced hemagglutination 8 to 64 times more effectively than α‐methylsialoside.^[^ ^16^ ^]^ To investigate whether an aromatic group at the C‐2 position of the sialoside could similarly enhance binding to SARS‐CoV‐2, we synthesized a polysialoside derivative dPG_100_(SA_aryl_)_0.20_ bearing this modification. This compound is structurally analogous to dPG_100_SA_0.20_ and was prepared as shown in the **Scheme** **1**. All compounds were thoroughly characterized by spectroscopic techniques. Successful conjugation of sialosides or sulfates was determined by ^1^HNMR and elemental analysis. The ζ‐potentials and hydrodynamic diameters (D_h_) of the polymers were determined in phosphate buffer (10 mm, pH 7.4) (**Table** **1**, Figures S17 and S18, Supporting Information for synthesis and characterization). Further physicochemical properties of different polymer nanoparticles are given in the Table 1.

**Table 1:** Characterization of functionalized dPG_500_ and dPG_100_ and non‐functionalized controls.

Compound^a)^ (PG_MW_Ligand_DF_)	SA or OSO_3_Na/NP^b)^	DF^c)^ [%]	*D_h_* ^d)^ [nm]	PDI^d)^	SA or OSO_3_Na/nm^2^ ^e)^	ζ‐potential ± SD^f)^ [mV]
dPG_500_	–	–	13.21 ± 0.41	0.35	–	−6.91 ± 1.67
dPG_500_SA_0.25_	1824	27	14.61 ± 0.18	0.17	2.72	−28.2 ± 1.44
dPG_500_SA_0.55_	3648	54	13.89 ± 0.21	0.43	6.10	−45.9 ± 2.76
dPG_500_C_0.20_	1300	20	14.02 ± 0.39	0.56	2.11	−22.1 ± 6.94
dPG_500_S_0.25_	1625	25	13.33 ± 0.25	0.39	2.88	−26.2 ± 6.47
dPG_500_S_0.55_	3575	55	11.40 ± 0.17	0.59	8.76	−36.3 ± 9.67
dPG_500_SA_0.20_S_0.20_	2600	40	12.83 ± 0.17	0.53	2.52	−48.7 ± 7.64
dPG_100_	–	–	10.18 ± 0.64	0.41	–	−6.68 ± 0.18
dPG_100_SA_0.20_	297	22	9.60 ± 1.12	0.59	1.02	−38.8 ± 0.95
dPG_100_(SA_aryl_)_0.20_	297	22	10.29 ± 0.62	0.57	1.02	−14.6 ± 2.78

^a)^ Polymer structure is indicated by the molecular weight (MW) of the dendritic polyglycerol (dPG) backbone and the degree of functionalization (DF) of either sialic acid (SA), sulfate (S) or carboxylic acid groups (C);

^b)^ Number of SA units per polymer, calculated from DF as determined by ^1^HNMR. DF is the percentage of total OH groups on dPG that were functionalized with the respective ligands;

^c)^ Determined by ^1^HNMR analysis;

^d)^ Determined with DLS, measured in aqueous buffer solution (PB, pH 7.4), mean values of triplicates ± standard deviation of the volume distribution profile;

^e)^ Average SA densities, on the surface of an assumed spherical dPG particle, calculated by the determined number of SA;

^f)^ Determined surface potential by measuring triplicates of the zeta potential in aqueous buffer solution (PB, 10 mm, pH 7.4) together with standard deviation.

### 2.2 Affinity Characterization of Polymer Nanoparticles Against SARS‐CoV‐2 Spike Proteins or Whole Virus Particles

**Figure 2:**
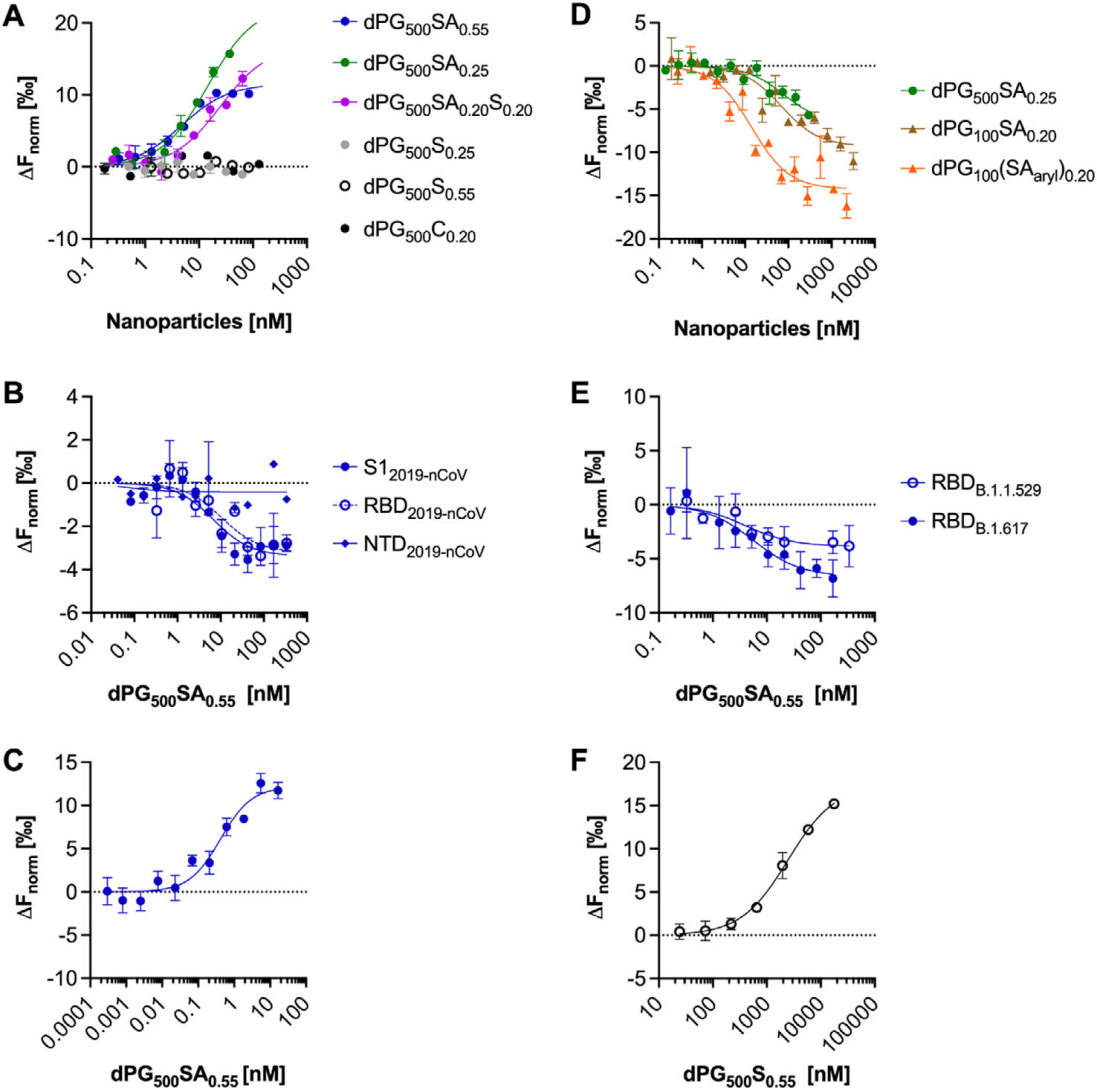
Affinity measurements using microscale thermophoresis with **A)** SARS‐CoV‐2 wild‐type variant B.1 (WT, D614G) (full virus) against dPG‐conjugates; **B)** Domains of the Spike protein from the wild‐type variant against dPG_500_SA_0.55_; **C)** SARS‐CoV‐2 variant B.1.617 (Delta) (full virus) against dPG_500_SA_0.55_; **D)** RBD protein from wild‐type variant against dPG_500_SA_0.25_ with a 500 kDa backbone, dPG_100_SA_0.20_ with a 100 kDa backbone and dPG_100_(SA_aryl_)_0.20_; **E)** dPG_500_SA_0.55_ against RBD proteins from B.1.617 (Delta) and B.1.1.529 (Omicron) variants; **F)** SARS‐CoV‐2 wild‐type variant B.1 against dPG_500_S_0.55_ at higher nanoparticle concentrations. In dilution series of the compounds, binding with spike proteins or virus particles could be detected upon a change in fluorescence (ΔF_norm_) between the initial fluorescence before start of the infrared laser (IR) and 1.5 s after the start of IR, inducing a thermophoretic gradient. The data were normalized by subtracting the background fluorescence. In A‐F each data point represents biological repeats of N = 3 except for NTD_2019‐nCoV_ (Figure 2B, N = 2), dPG_500_SA_0.20_S_0.20_ (Figure 2A, N = 5), RBD_B.1.617_ (Figure 2E, N = 4). Mean values are given together with standard error of the mean (SEM). Data points were fitted with a one‐sided fit assuming a 1:1 ligand to receptor ratio. Binding constants are described as apparent dissociation constants (Kdapp_NP_) assuming a 1:1 binding stoichiometry for viruses or proteins and scaffolds (see Table 2). The resultant data were plotted with Prism 9.2 (GraphPad by Dotmatics).

In order to determine the affinities of the synthesized nanoparticles, we used MST technique that measures binding affinity between molecules by detecting changes in the movement of a fluorescently labeled target (e.g., protein or virus) within a microscopic temperature gradient.^[^ ^17^ ^]^ Upon binding to a ligand, the size, charge, or hydration shell of the labeled molecule changes, leading to a shift in its thermophoretic behavior. These changes are quantified as normalized fluorescence (ΔFnorm), which is plotted against nanoparticle concentration to generate binding curves. We demonstrated earlier that such binding measurements can also be performed with whole Influenza A virus particles.^[^ ^13^ ^]^ These measurements allow the quantification of multivalent binding events in equilibrium by introducing an apparent dissociation constant (K_d,app_). We first measured binding of the different polymers against whole SARS‐CoV‐2 B.1 (WT, D614G) particles. We found that polymers functionalized with SA i.e. dPG_500_SA_0.25 or 0.55,_ dPG_100_SA_0.20_ or its aromatic variant dPG_100_(SA_aryl_)_0.20_ with nm K_d_ values had much stronger (≈up to 500 times) binding compared to the high‐density sulfated versions dPG_500_S_0.55_ with K_d_ of 2.46 µm (**Figure** **2**A, Table 1). The µm K_d_ values observed for polysulfates are in agreement with an earlier investigation reported by Nie et al., in which high‐density dendritic sulfated polyglycerol exhibited K_d_ of 144 µm against the RBD of wild‐type SARS‐CoV‐2.^[^ ^8^ ^]^ Interestingly, the heteromultivalent dPG_500_SA_0.20_S_0.20_ showed with a K_d_ of 24.92 nm a similar binding affinity compared to the homomultivalent sialoside dPG_500_SA_0.25_ with only 2‐fold lower K_d_ (10.85 nm) (Table 1). This indicates that statistically distributed sulfates in addition to sialosides on the dPG backbone did not strongly enhance binding to SARS‐CoV‐2 particles. Also, dPG_500_SA_0.25_ showed 8 times stronger binding to the wildtype virus compared to wildtype RBD indicating multivalent binding effect. Furthermore, the polycarboxylated analog dPG_500_C_0.20_ did not show binding with the SARS‐CoV‐2 B.1 (WT, D614G) indicating that sialic acid interactions with the SARS‐CoV‐2 are not merely electrostatic. Notably, introducing aromaticity on SA at the anomeric C2 position improved binding by a factor of about five, as demonstrated by dPG_100_(SA_aryl_)_0.20_ having a lower dissociation constant (K_d_ 14.22 nm) compared to dPG_100_SA_0.20_ (K_d_ 69.14 nm) (Figure 2D). All obtained values are listed in **Table** **2**.

**Table 2:** Summary of apparent dissociation constants (*K*_d, app, NP_) determined from microscale thermophoresis (MST) experiments. The number of biological repeats for each binding interaction is N = 3, except for NTD_2019‐nCoV_ vs dPG_500_SA_0.55_ (N = 2), full wild‐type virus vs dPG_500_SA_0.20_S_0.20_ (N = 5), RBD_B.1.617_ vs dPG_500_SA_0.55_ (N = 4). Mean values are given together with standard error of the mean (SEM). Kd, app values were derived from experiments shown in Figure 2, by using a one‐site special fit function. n.d. not detectable until 10 µm nanoparticles.

Compound (PG_MW_Ligand_DF_)	*K*_d,app_ [nm]						
	Wild‐type (full virus) B.1 (WT, D614G)	S1 (WT)	RBD (WT)	NTD (WT)	Delta (full virus) B.1.617	RBD Delta B.1.617	RBD Omicron B.1.1.529
dPG_500_SA_0.55_	4.78 ± 1.19	3.14 ± 2.89	7.15 ± 7.31	n.d.	0.42 ± 0.16	5.70 ± 1.82	3.98 ± 1.45
dPG_500_SA_0.25_	10.85 ± 2.65	–	80.11 ± 47.67	–	–	63.04 ± 51.20	–
dPG_100_SA_0.20_	61.80 ± 26.64	–	69.14 ± 32.57	–	–	–	–
dPG_100_(SA_aryl_)_0.20_	9.01 ± 13.97	–	14.22 ± 32.57	–	–	–	–
dPG_500_S_0.55_	2466 ± 250.34	–	–	–	–	–	–
dPG_500_SA_0.20_S_0.20_	24.92 ± 5.75	–	–	–	–	–	–
dPG_500_C_0.20_	n.d.	–	–	–	–	–	–
dPG_500_S_0.25_	n.d.	–	–	–	–	–	–
6’ – sialyllactose		–	n.d.	–	–	–	–

Based on these findings, we further probed the binding of the nanoparticles to defined domains of the S‐protein of wild‐type SARS‐CoV‐2. First, we characterized the binding of nanoparticle to the recombinant RBD of the wild‐type SARS‐CoV‐2 (2019‐nCoV), which is known to interact with ACE2, heparan sulfate (HS) and a proposed SA binding site. By testing polysialosides with significantly different SA densities, we found that dPG_500_SA_0.55_ (K_d_ 7.15 nm) enhanced the binding affinity to RBD [SARS‐CoV‐2 (2019‐nCoV)] by approximately 11‐fold compared to scaffolds with lower SA density dPG_500_SA_0.25_ (K_d_ 80.11 nm), respectively. No significant binding was observed to the recombinant NTD protein of the wild‐type variant SARS‐CoV‐2 (2019‐nCoV) (Figure 2B, Table 2). Finally, multivalent dPG_500_SA_0.55_ bound equally effectively to the RBD protein of the Omicron variant (B.1.1.529) and had 14 times improved binding affinity to the full virus Delta variant (B.1.617) (Figure 2C,E, Table 2).

### 2.3 Theoretical Analysis: MD Simulation and Ensemble Docking

**Figure 3:**
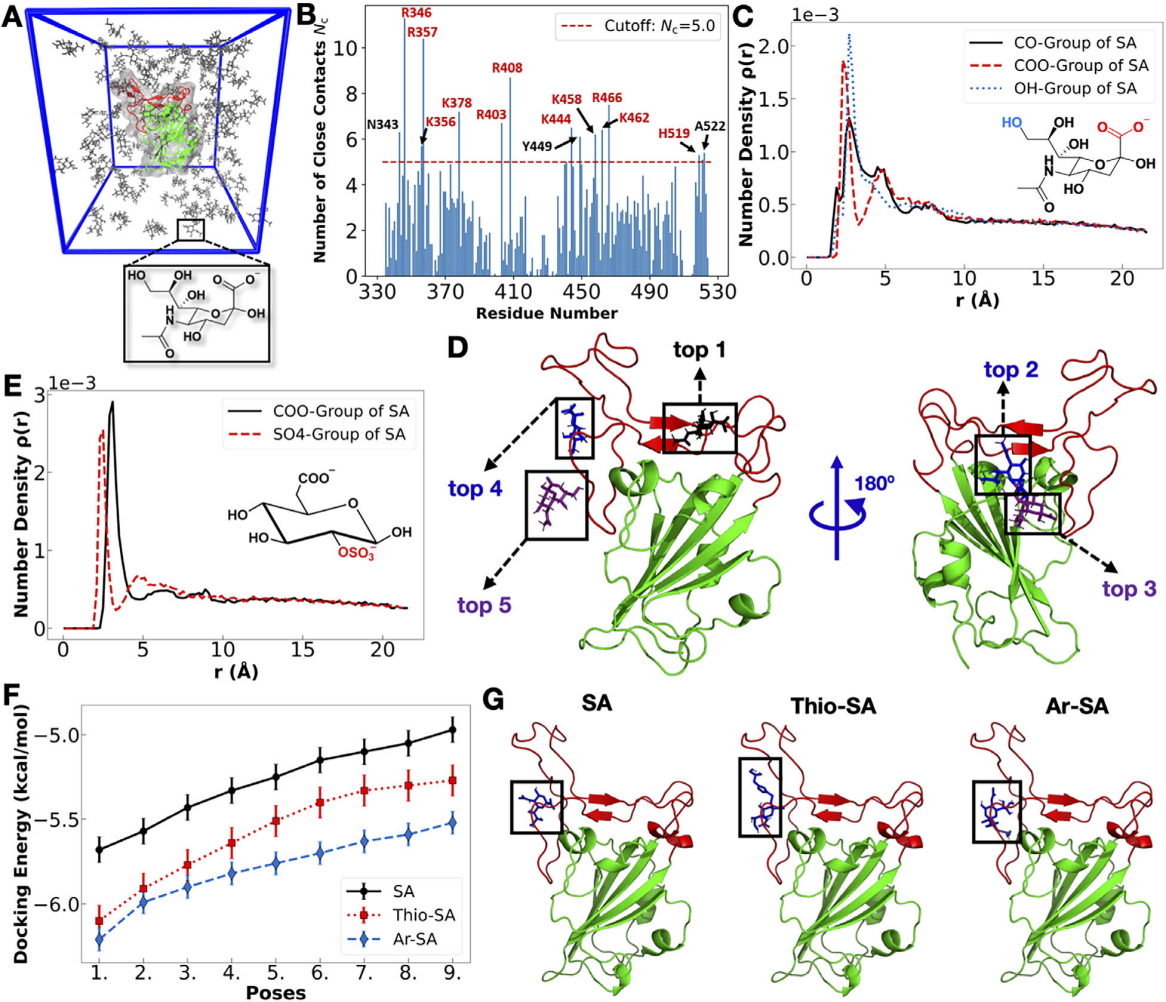
A) Simulation unit cell containing a single RBD (in green except the receptor binding motif, RBM, in red) and sialic acid, SA, monomers (in gray). The chemical structure of SA is shown below the simulation box. Water and ions are present in the simulation box but not shown for clarity. B) Number of close contacts *N_c_* between SA ligands and different amino acid residues of RBD (averaged over simulation time of 1000 ns). Residues having *N_c_* > 5 are labeled, cationic residues in red and charge neutral residues in black. C) Number density distribution of different functional groups (‐CO, ‐COO^–^, ‐OH) of SA ligands around RBD as a function of the distance *r* from the RBD surface. D) Snapshots of binding poses of SA obtained from the top five longest residing ligands near the RBD surface in the MD simulation (for the details on the residue‐level interactions, see Figure S19 in the Supporting Information). SA ligands shown in black, blue, or violet. RBD is shown in green except RBM (amino acid residues 438–506) in red. E) Number density distribution of different functional groups (‐COO^–^, ‐SO_4_^–^) of BGLC ligands around RBD as a function of *r*. The chemical structure of BGLC is shown in the inset. F) Docking interaction energies of different functionalized sialic acids (SA, Thio‐SA, Ar‐SA) with RBD for the top nine docking poses. Each data point and the bar represent the average value and the standard error of ten different ligand‐docking studies taking different RBD conformations selected from the simulation of RBD and SA ligands. G) Snapshots of the best docking poses (with the RBD structure extracted from the simulation in the water‐only solution after 400 ns) for the different functionalized SAs (marked by rectangles and names provided on the top). RBD representation is the same as in the panel D. For the details on the residue‐level interactions, see Figure S20 in the Supporting Information.

To understand the relevance of different functional groups of dPGs, we have performed explicit‐solvent all‐atom MD simulations of the SARS‐CoV‐2 spike protein RBD in solutions of different ligands. We have considered only the RBD in the simulations, as it is inferred from the MST measurements that sialylated dPGs predominantly interact with the RBD (Table 2). Though earlier experimental and simulation studies have suggested that SAs bind both to the NTD^[^ ^18^, ^19^, ^20^, ^21^ ^]^ and the RBD,^[^ ^22^, ^23^, ^24^, ^25^ ^]^ SAs grafted to dPGs can form multivalent interaction with the RBD because of its larger solvent‐accessible surface area, especially in the up conformation. In addition, the NTD surface compared to the RBD, is highly shielded by glycans,^[^ ^26^ ^]^ restricting its multivalent interaction with sialylated dPGs. For the functionalization with sulfate groups, our earlier studies have revealed that polysulfates interact mostly with the cationic patch on the RBD.^[^ ^8^, ^27^ ^]^ The simulation unit cell is shown in **Figure** **3**A and details of the simulation method and data analysis are presented in the supporting information (SI). We observe that the sialic acid monomer, i.e., N‐acetylneuraminic acid binds to the RBD via multiple binding modes, snapshots for the top five binding poses are shown in Figure 3D. The number of close contacts plot reveals that despite SA binding to different types of surface residues of RBD, it forms a greater number of contacts with cationic amino acids (Figure 3B). The number density plot, however, shows that not only the anionic carboxylate group but also charge‐neutral hydroxyl and carbonyl groups of SA have similar propensities toward the RBD (Figure 3C). Because of the additional interactions formed by SA, multivalent binding of polysialosides with one RBD can be formed. This could be rationalized based on the decrease in K_d_ values with increasing the degree of sialylation (see Table 2), which cannot be explained by a 1:1 SA:RBD ratio.

To understand whether the experimentally determined enhanced binding affinity of sialylated dPGs to the RBD, compared to sulfated ones, is due to only the carboxylate of SA or its additional functional groups as well, we check the competitive binding between carboxylate and sulfate groups by performing MD simulation of the RBD in a solution of BGLC (a derivative β‐D‐Glucose, taken from a Heparin monomer, with both carboxylate and sulfate groups) ligands. Number density profiles for these two groups around RBD show a slightly greater number of carboxylates present near RBD, compared to sulfates (Figure 3E). However, analysis of the residence time τ, i.e. the average duration for which a ligand stays within a close proximity to the RBD, indicates that the sulfate group resides twice longer than carboxylate, with τ  =  12 ns for COO^–^ and τ  =  22 ns for SO_4_^–^ (see Figure S21 and the discussion in the Supporting Information). Since the residence time is inversely proportional to the exponential of the binding free energy Δ*G_b_* (with Δ*G_b_* < 0), we estimate from the τ values that compared to carboxylate, the sulfate group binds to RBD with an additional free energy gain of ‐0.61 k_B_T (see calculation details in the Supporting Information). Thus, other functional groups of SA apart from the carboxylate, as shown in Figure 3C, contributes significantly to strengthening the binding of SA to RBD. This finding further supports the hypothesis that the RBD contains binding sites for SA.

The MST measurements indicated that the linking groups at the anomeric position of SA (Scheme 1) influenced sialylated dPGs’ dissociation constant, *K_d_*, values and hence their binding free energy, Δ*G_b_*, values since both are related as Δ*G_b_* =  *RT*ln (*K_d_*/*c*_0_), where *R* is the ideal gas constant, *T* represents temperature, and the standard‐state concentration *c*_0_ =  1 mol/L. In particular, including an aryl group in the anomeric position of SA leads to a decrease in *K_d_* and thus an increase in the binding affinity. To understand whether this arises from direct, favourable interactions of the aryl group with RBD or some other subtle effects, we have performed ensemble docking studies (details provided in the Supporting Information) for SA, the ‐S‐triazolyl and ‐O‐aryl substituted SAs used in experiments. We found that the magnitude of the docking interaction energy of aryl substituted SA (Ar‐SA) is higher than the thio‐triazolyl functionalized SA (Thio‐SA) for the top nine docking poses (Figure 3F). Compared to SA, both Ar‐SA and Thio‐SA interact more strongly with RBD because of their additional functional groups. Interestingly, all three variants of SA bind to the receptor binding motif (RBM), the part of RBD that forms direct contact with the ACE2 receptor protein on the host cell, as seen from the best docking poses in Figure 3G. The SA binding sites obtained from the ensemble docking study match with “top 4” and “top 5” binding poses of SA obtained from the MD simulation (Figure 3D). However, the top 1–3 SA binding poses seen in the MD simulation, a much more realistic method combined with an adequate sampling time of 1 µs, are not observed in any of the binding poses obtained from the ensemble docking study. A detailed discussion of ligand binding sites on RBD for the different types of SAs is provided in the Supporting Information SI, and the RBD residues involved in the binding are given in Tables S2 and S3 in the Supporting Information. Note that though our RBD model excludes glycosylations, this is not expected to affect the observed SA binding sites, as the glycosylation sites are far away from the RBM.^[^ ^26^, ^28^ ^]^

### 2.4 SARS‐CoV‐2 Replication And Entry Inhibition in Calu‐3 Cells

After finding dPG_500_SA_0.55_ as the high affinity ligand for the SARS‐Cov‐2, we next tested the potential of synthesized sialylated compounds for SARS‐CoV‐2 B.1 (WT, D614G) infection inhibition of human lung derived Calu‐3 cells.

**Figure 4:**
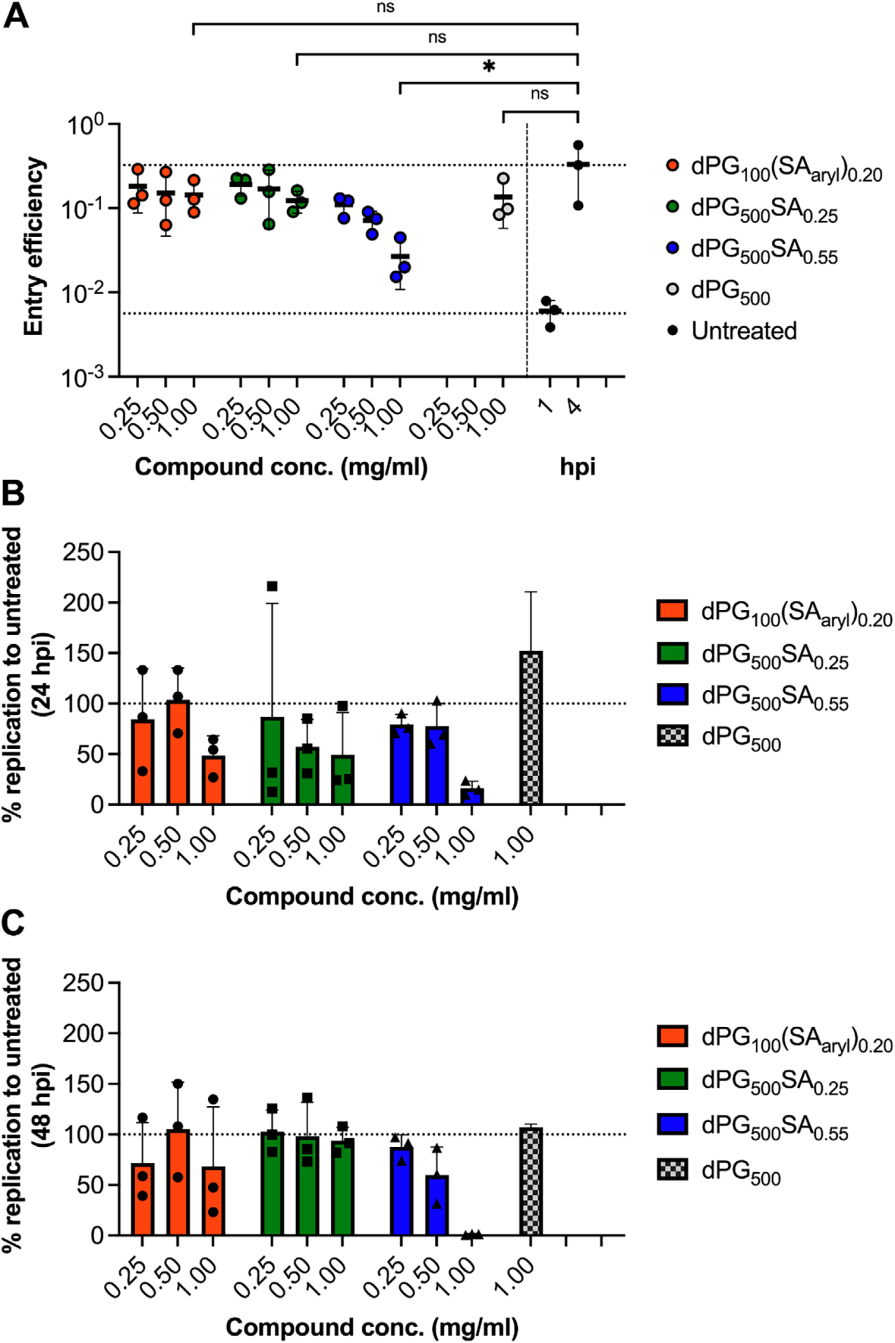
SARS‐CoV‐2 replication and entry in Calu‐3 cells is inhibited by dPG_500_SA_0.55_. A) Calu‐3 cells were pre‐ and post‐treated with 0.25, 0.5, or 1 mg ml^−1^ of the indicated compounds before cells were infected with SARS‐CoV‐2 (MOI 2) at 4 °C to allow synchronized entry. Entry efficiency was determined from cell lysates at 4 hpi with a highly sensitive quantitative RT‐PCR for nucleocapsid‐specific subgenomic RNA. Entry efficiency was calculated by the delta ct method and by using the expression of cellular TATA‐binding protein (TBP) as a reference. Upper dotted line represents the mean virus entry efficiency at 4 hpi and the lower dotted line represents the mean virus entry efficiency at 1 hpi. Data show means of three independently conducted experiments each performed in triplicates. B,C) Calu‐3 cells were infected with SARS‐CoV‐2 (MOI 0.001) and pre‐ and post‐treated with 0.25, 0.5 or 1 mg ml^−1^ of the indicated compounds. Virus replication was determined with an envelope‐specific quantitative RT‐PCR at 24 hpi; B) and 48 hpi; C) from the supernatant of infected cells. Dotted lines represent % virus replication in untreated samples. The data was plotted using GraphPad Prism 10, displaying individual data points along with mean values of three technical repeats. Error bars indicate standard deviation (SD). Statistical significance was determined by an ordinary one‐way ANOVA followed by a Dunnett's multiple comparison test (confidence level 95%), referring to the control group (4 hpi): *p < 0.0332, **p < 0.0021, ***p < 0.0002, and ****p < 0.00001. Conc.: concentration; hpi: hours post infection; ns: not significant.

We first investigated whether synthesized polysialosides can also block entry of authentic SARS‐CoV‐2 virions. To determine entry efficiency, Calu‐3 cells were infected with SARS‐CoV‐2 at 4 °C to allow synchronized entry, while cells were pre‐ and post‐treated with increasing concentration of compounds. Nucleocapsid‐specific subgenomic RNA is produced during coronavirus infection early after entry in high quantities^[^ ^29^ ^]^ and was applied to compare the entry efficiency of SARS‐CoV‐2 upon compound treatment. Only dPG_500_SA_0.55_ inhibited SARS‐CoV‐2 entry significantly to 18.6% at 0.5 mg ml^−1^ and of 54% at 1 mg ml^−1^, when compared to dPG_500_ control treated Calu‐3 cells (**Figure** **4**A).

In the next step, the biological assay was set up to determine if these compounds inhibit authentic SARS‐CoV‐2 replication. Calu‐3 cells were infected with SARS‐CoV‐2 isolate in the presence of increasing inhibitor concentrations, which were supplied before infection and for the entire duration of the experiment. In the presence of dPG_500_SA_0.55_ SARS‐CoV‐2 replication was inhibited up to 83.8% at 24 hours post‐infection and to 98.9% at 48 hours post‐infection at the maximum applied compound concentration of 1 mg ml^−1^ which is equivalent to 0.5 µm, when compared to untreated Calu‐3 cells (Figure 3B,C). The low‐density sialylated dPG_500_SA_0.25_ and aromatically modified sialylated dPG_500_(SA_aryl_)_0.20_ analogs showed only very weak inhibition at 24 hpi at the highest concentrations applied. A high density of SA seems to be important for virus infection inhibition. The control compound without any SA, dPG_500_ did not show any inhibition.

A cell viability assay was conducted to exclude the possibility that the compounds were cytotoxic. The number of viable cells remained at a constant level with increasing compound concentration at the highest dose of 1 mg ml^−1^ after 24h and 48h post treatment (Figure S23, Supporting Information). This confirms the specific action of the compounds.

In summary, dPG_500_SA_0.55_ was identified as a SARS‐CoV‐2 entry inhibitor in Calu‐3 cells, which presumably blocks the attachment of virions to cells before specific interaction with cellular receptors occurs.

### 2.5 Cryo‐TEM Analysis

**Figure 5:**
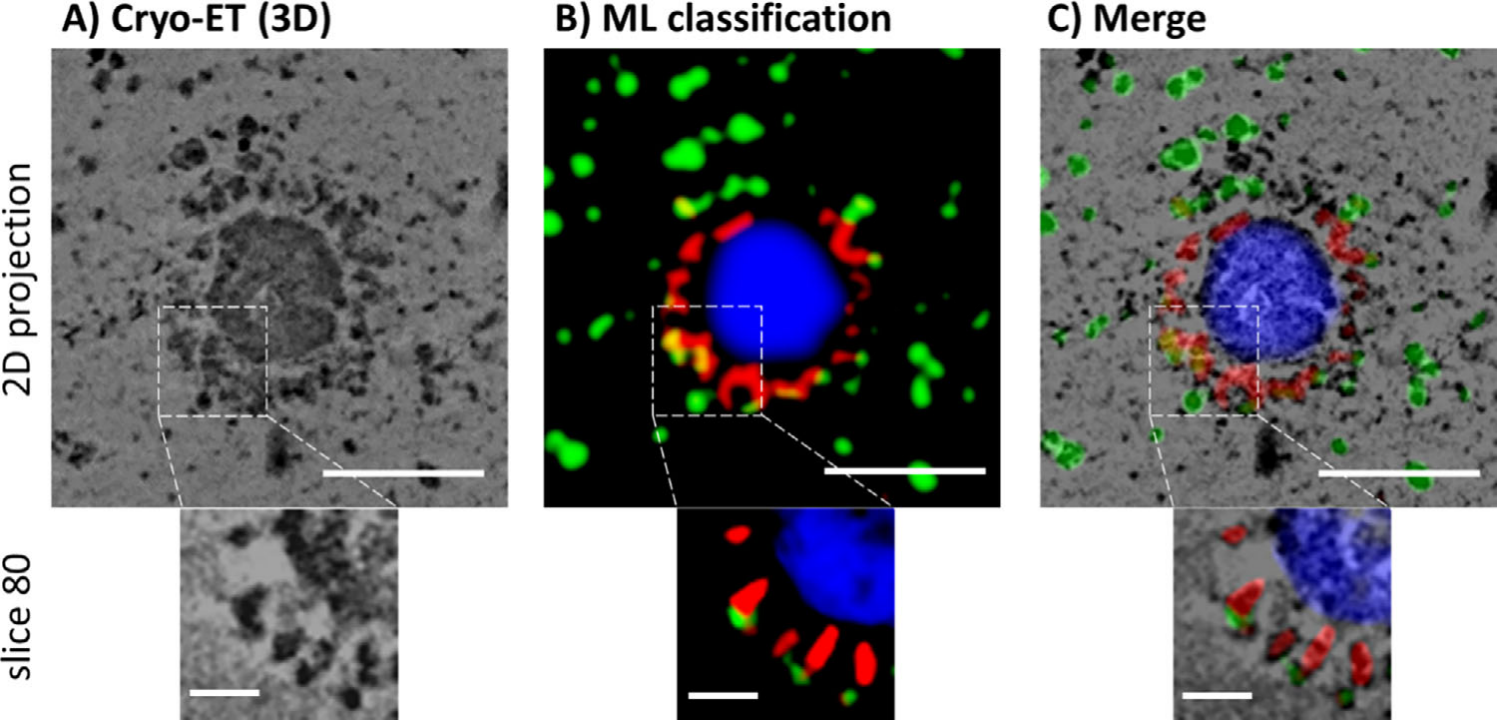
Cryo‐ET visualization and machine learning‐based segmentation of dPG_500_SA_0.55_ nanoparticles binding to SARS‐CoV‐2 spike protein RBDs. The top panels show a 2D projection of the 3D tomogram and the bottom panels show a zoom of one in‐plane slice of the 3D tomogram. A) 3D reconstruction obtained from a recorded cryo tilt series. B) Segmented 3D volume: To distinguish between the S head domains (S1) of the spike proteins (the flexible and thin S2 stem is mostly not visible in the 3D reconstruction due to limited resolution) and the sialylated dPG cores, the trainable Weka (Waikato Environment for Knowledge Analysis) segmentation classifier was applied. dPG nanoparticles (green), spike proteins (red), and virus body (blue) highlighted). **C)** Overlay of the original image with the segmented representation. Top scale bars: 100 nm. Bottom scale bars: 25 nm.

To visualize the binding of sialylated dPG compounds to the S‐proteins of SARS‐CoV‐2, we cryo‐prepared the ligand with the highest affinity, i.e., dPG_500_SA_0.55_ together with the virus particles by plunge freezing into liquid ethane to obtain a snapshot of the conditions under hydrated conditions and to analyze subsequently the viruses embedded in the amorphous ice using cryo‐electron transmission microscopy (cryo‐TEM). However, to ensure that the TEM projection images did not simply show overlays of virions and dPG_500_SA_0.55_, cryo‐electron tomography (cryo‐ET) combined with machine learning‐based segmentation was used (**Figure** **5**).

Figure 5A shows the 3D reconstruction obtained from a recorded cryo tilt series. To distinguish between the S head domains (S1) of the spike proteins (the flexible and thin S2 stem is mostly not visible in the 3D reconstruction due to limited resolution) and the sialylated dPG cores, we used the machine learning algorithms of the trainable Weka (Waikato Environment for Knowledge Analysis) segmentation (more details in the Supporting Information). The Segmentation was done in Fiji.^[^ ^30^ ^]^ Figure 5B shows the resulting overlay of cryo‐ET 3D and classified structures (green: dPG_500_SA_0.55_, red: spike proteins, blue: virus core).

The segmented image (panel C) reveals a clustering of nanoparticles (green) around the virus particle, particularly in areas rich with spike proteins (red). The yellow regions, indicating an overlap between red and green signals, indicate direct interactions between the nanoparticlesand SARS‐CoV‐2 spike proteins. This colocalization pattern demonstrates that the sialoside‐functionalized dPG nanoparticles specifically bind to viral spike proteins.

## 3 Conclusions

This study demonstrates that a synthetic polysialoside can inhibit SARS‐CoV‐2 infection by direct binding with RBD on the S1 spike protein. The MD simulation, ensemble docking, and MST studies show that the direct binding of SA with RBD is not merely electrostatic but SA as a whole sugar molecule has a role in the binding interactions. The synthetic polysialoside binds at low nm concentrations (K_d_ = 4.78 nm) in contrast to a polysulfated analog that binds at µm concentrations (K_d_ = 2.46 µm). The study also outlines that high density of SA on the dendritic polymer is crucial for the SARS‐CoV‐2 post‐infection inhibition. Infection inhibition tests performed at different time points indicate that SARS‐CoV‐2 infection drops dramatically by inhibiting the virus entry into Calu‐3 cells in the presence of polysialoside. Overall, these findings demonstrate that high‐density polysialoside represents a promising therapeutic strategy against SARS‐CoV‐2 infection through its nanomolar binding affinity to the RBD and effective inhibition of viral entry.^[^ ^31^ ^]^ Further biological studies in the BSL3 laboratory, comparing different functionalities conjugated to polymer nanoparticles across various strains of SARS‐CoV‐2, will yield valuable insights.

## 4 Experimental Section

### Microscale Thermophoresis Measurements (MST)

Binding affinities were determined via microscale thermophoresis (MST) using the Monolith NT.115 instrument with premium capillaries (SKU: MO‐K025), both from Nanotemper. All proteins used in this study were ordered from Sino Biological and labeled using the protein labeling kit Red‐NHS 2^nd^ generation (SKU: MO‐L011) from Nanotemper following the provided protocol. The proteins used in this study were SARS‐CoV‐2 (2019‐nCoV) Spike RBD (40592‐V08H), SARS‐CoV‐2 (2019‐nCoV) Spike S1 (40591‐V08H), SARS‐CoV‐2 (2019‐nCoV) Spike NTD (40591‐V49H), SARS‐CoV‐2 (2019‐nCoV) Spike RBD (L452R, E484Q) (40592‐V08H88), SARS‐CoV‐2 B.1.1.529 sublineage BA.2 (Omicron) Spike RBD (40592‐V08H121). The binding affinities of full SARS‐CoV‐2 viruses of wild‐type (Wuhan) and delta variants were also measured and the viruses were labeled using Octadecyl Rhodamine B Chloride (R18) fluorescent dye. Briefly, the virus was incubated with dye at room temperature in dark conditions for 30 minutes before the excess dye was removed using the Zeba Spin Desalting Column (7K MWCO) from Thermo Fisher Scientific (89883) following the manufacturer's protocol. The final concentrations of the labeled proteins and the labeled viruses were determined using a Nanodrop instrument (Thermo Fisher Scientific). The MST protocol was adapted from Bhatia *et al* 2017^[^ ^13^ ^]^ the tested polymers were serially 2‐fold diluted in DPBS with 0.05% (v/v) Tween 20 (PBST) with a resulting volume of 4 µL. 4 µL of labeled proteins at a concentration of 5 nm was added to the mixture resulting in a final volume of 8 µL. For labeled virus PBS without Tween 20 was used. The final mixture was shortly equilibrated at room temperature before collecting the mixtures in premium capillaries for measurement. MST measurements were performed at 25 °C for initial fluorescence (5 s) and MST over a 30 s time interval using the MO. Control software (Nanotemper) at 50% green‐LED excitation and 40% IR‐laser power with R18 labeled viruses and at 40% red‐LED excitation and 40% IR‐laser power with Red‐NHS labeled proteins. The apparent dissociation constant (K_d,app_) was calculated from a one‐site saturation fit (Equation (1): assuming a 1:1 binding stoichiometry between the fluorescently labeled virus or virus protein and the polymer).

(1) $$Y=\mathrm{Bma}x^{*}X/\left(K_{d}\;+\;X\right)$$

- X: Concentration of the radioligand
- Y: Specific binding
- Bmax: Maximum binding, expressed in the same units as Y
- Kd: Expressed in the same units as X

The resultant data were analyzed via Affinity Analysis Software v2.3 (Nanotemper), exported, and plotted with Prism 9.2 (GraphPad by Dotmatics).

### Molecular Dynamics Simulations

The starting structure of the receptor binding domain (RBD) of the SARS‐CoV‐2 spike protein was obtained from the protein database (PDB ID: 6M0J). An MD simulation was performed for a single RBD in a solution of sialic acid, N‐acetyl‐neuraminic acid (SA) ligands. The SA ligand was built by using the CHARMM‐GUI *Glycan Reader & Modeler*.^[^ ^32^ ^]^ Force field parameters for SA were derived from the CHARMM general force field^[^ ^33^, ^34^ ^]^ using the CGenFF program.^[^ ^35^, ^36^ ^]^ CHARMM36m force field parameters (March 2019)^[^ ^37^ ^]^ were used for the RBD. The RBD and SA ligands were solvated in a tetragonal box of size 8.0×8.0×9.0 nm. To achieve a ligand concentration of 0.5 M, a total of 139 SA ligands were added. The system was solvated with 15447 water molecules. 139 Na^+^ ions and 2 Cl^−^ ions were added to charge‐neutralize the system. The CHARMM‐compatible TIP3P water model^[^ ^38^ ^]^ and ion parameters^[^ ^39^ ^]^ were used.

The MD simulation was performed using the Gromacs simulation package (version 2020.6).^[^ ^40^ ^]^ First, bad contacts present in the initial structure of the system were removed by energy minimization steps using the steepest descent algorithm, until the maximal force on individual atom becomes smaller than 1.0 kJ mol nm^−1^. Then, a 300 ps equilibration run in the NVT ensemble followed by an equilibration run of 300 ps and a production run of 1000 ns were performed in the NPT ensemble. The backbone atoms of the protein residues 333, 416, 526 were frozen throughout the simulation to stop center‐of‐mass translation and rotation of the RBD. The temperature was kept constant by using the stochastic velocity rescaling algorithm^[^ ^41^ ^]^ with a coupling time constant of 0.1 ps and a reference temperature of 300 K. The pressure was controlled using an isotropic Parrinello‐Rahman barostat^[^ ^42^ ^]^ with a coupling time constant of 2 ps and a reference pressure of 1.0 bar. The simulation was performed with periodic boundary conditions applied in all 3 directions (x, y, and z). The LINCS^[^ ^43^ ^]^ algorithm was employed to constrain the bonds involving H‐atoms, and a timestep Δt = 2 fs was used. The particle mesh Ewald summation method^[^ ^44^ ^]^ with a real‐space cutoff distance of 1.2 nm was used for the calculation of long‐range electrostatics interactions, while for the short‐range van der Waals interactions, the Lennard‐Jones potential was used with a cutoff distance of 1.2 nm and the resulting forces smoothly switched to zero between of 1 nm to 1.2 nm.^[^ ^8^ ^]^ The output trajectories were saved at intervals of Δt = 10 ps for the purpose of subsequent analysis.

### Ensemble Docking Studies

Ensemble docking studies of SA, Thio‐SA, and Ar‐SA (see Scheme S2 for the chemical structures) with RBD were performed using AutoDock Vina (version 1.1.2).^[^ ^45^ ^]^ The AutoDock score was a linear combination of van der Waals, hydrogen bonding, electrostatics, and desolvation energy terms. A lower AutoDock score signifies stronger binding. The structure of SA was taken from the conducted MD simulation. Thio‐SA and Ar‐SA were built using the Avogadro software.^[^ ^46^ ^]^ In order to account for the diverse conformations of the RBD, ten different RBD conformations were extracted from the MD simulation trajectory of RBD in the water‐only solution (no ligands) at a time interval of 100 ns and ten separate docking studies were conducted, each utilizing a distinct RBD conformation. The MGLTools package was used to prepare the protein and ligand structures in the “.pdbqt” format. A blind docking approach, involving the docking of a ligand to the entire RBD surface, was performed with a rigid RBD protein and a flexible ligand. A box size of 7 × 7 × 7 nm^3^ was employed. To ensure thorough exploration of the binding conformational space, an exhaustiveness value of 200 was utilized. The maximum energy difference between binding modes was set to 6 kcal mol^−1^, and the maximum number of binding modes was set to 10. The protein‐ligand interaction profiler (PLIP)^[^ ^47^ ^]^web tool was used to examine residue‐level interactions between the different types of SA ligands and the RBD in the best docked conformations, as shown in Figure S20 (Supporting Information).

### X‐ray Photoelectron Spectroscopy (XPS)

XPS experiment was performed with an EnviroESCA spectrometer (SPECS Surface Nano Analysis GmbH, Berlin, Germany), equipped with a monochromatic Al Kα X‐ray source (Excitation Energy = 1486.71 eV) and a PHOIBOS 150 electron energy. Samples for XPS analysis were prepared on indium foil. The spectra were measured in normal emission, and a source‐to‐analyzer angle of 55° was used. All spectra were acquired in fixed analyzer transmission (FAT) mode. The binding energy scale of the instrument was calibrated, following a technical procedure provided by SPECS Surface Nano Analysis GmbH (calibration was performed according to ISO 15472). For quantification, the survey spectra were acquired at ultra‐high vacuum conditions (*p* < 1 × 10^−5^ mbar) with a pass energy of 100 eV, and the spectra were quantified utilizing the empirical sensitivity factors that were provided by SPECS Surface Nano Analysis GmbH (the sensitivity factors were corrected with the transmission function of the spectrometer).

### Cells and Culture Conditions

Calu‐3 cells (HTB‐55) were maintained at 37 °C and 5% CO_2_ in a humidified atmosphere and cultured in Dulbecco's Modified Eagle's Medium (DMEM, ThermoFisher Scientific) supplemented with 10% fetal bovine serum (FBS, Thermo Fisher Scientific), 1% nonessential amino acids 100x concentrate (NEAA, Thermo Fisher Scientific), and 1% sodium pyruvate 100 mM (NaP, Thermo Fisher Scientific) and split twice a week. For seeding and cultivation, cells were washed with phosphate buffered saline (PBS, Thermo Fisher Scientific) and detached with 0.05% trypsin‐EDTA solution (Thermo Fisher Scientific).

### Virus Infection

Infection experiments were conducted with BetaCoV/Munich/ChVir984/2020 (B.1, EPI_ISL_406862). SARS‐CoV‐2 virus stock was sequenced by next‐generation sequencing to confirm the absence of minority variants. Infection experiments were performed under Biosafety Level 3 (BSL‐3) conditions with enhanced respiratory personal protection equipment. Calu‐3 cells were seeded at a density of 60,000 cells per ml in 96‐well plates one day prior to infection. For infection, virus stock was diluted in OptiPRO SFM (Thermo Fisher Scientific) serum‐free medium according to the desired MOI. For virus adsorption, 100 µL (96‐well) of virus master mix was added to the cells and incubated at 37 °C in a 5% CO_2_ atmosphere with 95% humidity. After 1 hour, virus dilutions were removed, cells were washed three times with PBS, and wells were refilled with DMEM infection medium (DMEM supplemented with 2% FBS, 1% NEAA, 1% sodium phosphate). To determine virus replication, supernatants were harvested at the indicated time points, and diluted in MagNA Pure 96 external lysis buffer (Roche, Penzberg, Germany) buffer before replication was determined by quantitative RT‐PCR.

### Cell Viability Assay

CellTiter‐Glo Luminescent Cell Viability Assay reagent (#G7571, Promega) was used according to the manufacturer's protocol. Briefly, 60,000 Calu‐3 cells were seeded into a 96‐well white microtiter plate (#CLS3610‐48EA, Sigma‐Aldrich) and treated with the indicated compounds as described previously. Cell viability was determined at 24 and 48 hours post‐treatment, respectively, after the addition of CellTiter‐Glo reagent using a multiplate reader (Synergy HTX, Biotek, Agilent).

### Isolation of Viral RNA and Quantitative Real‐Time RT‐PCR Assay

For isolation of viral RNA, 50 µL of supernatant was diluted in 300 µL of MagNA Pure 96 external lysis buffer (Roche, Penzberg, Germany). All samples were heat inactivated for 10 minutes at 70 °C prior to export from the BSL‐3. Isolation and purification of viral RNA was performed using the MagNA Pure 96 System (Roche, Penzberg, Germany) according to the manufacturers’ recommendations. Viral RNA was quantified using real‐time RT‐PCR (envelope gene assay) as previously described.^[^ ^48^ ^]^

### Synchronized Infection Experiments

Synchronized infection experiments were performed as previously described^[^ ^49^ ^]^ to determine the entry efficiency of SARS‐CoV‐2 upon compound treatment. Briefly, infection of cells was performed on ice, and cells were immediately transferred to 4 °C for 1 hour after virus dilutions were added to ensure synchronized virus uptake and start of replication. After virus adsorption, cells were washed 5 times with PBS to remove excess virus particles. Cells were lysed either immediately or incubated with infection medium until 4 hours post‐infection. At the indicated time points, medium was removed and cells were lysed with MagNA Pure 96 external lysis buffer (Roche, Penzberg, Germany). Isolation of RNA from cell lysates and quantitative RT‐PCR on subgenomic nucleocapsid RNA was performed as described elsewhere.^[^ ^49^ ^]^ A bead‐based method for RNA binding was utilized ensuring that any non‐nucleic acid components were effectively removed during the washing steps. Compounds were dissolved in PBS (pH 7.4, 10 mm) in the indicated concentrations, added 1 hour prior to virus infection, and were supplied for the entire duration of the experiment.

For quantitative RT‐PCR, a 12.5 µL reaction with 2.5 µL RNA was done with the SuperScript III 1‐step reverse transcriptase‐PCR system (Invitrogen) with the Platinum Taq DNA polymerase according to the manufacturers’ protocol and the following primers and probe: nCoV sgN Fwd: 5′‐CGA TCT CTT GTA GAT CTG TTC TC‐3′, nCoV sgN Rev: 5′‐CAG TAT TAT TGG GTA AAC CTT GG‐3′ and nCoV sgN prb: 5′‐56‐FAM/ CAG TAA CCA GAA TGG AGA ACG CAG /3BHQ‐1‐3′.^[^ ^50^ ^]^ The RT‐PCR was performed using a thermocycling protocol with reverse transcription for 15 minutes at 55 °C and a subsequent denaturation step for 2 minutes at 95 °C to restore Taq DNA polymerase activity, followed by PCR amplification by 45 cycles of 95 °C for 15 seconds and 58 °C for 30 seconds. Fluorescence signals were detected after the elongation step of each cycle. The mean fold change in gene expression was calculated, by the delta ct method, and by using the expression of TATA‐binding protein (TBP) as a reference.

### Statistical Analysis

The data was plotted using GraphPad Prism 10, displaying individual data points along with mean values of three technical repeats. Error bars indicate standard deviation (SD). Statistical significance was determined by an ordinary one‐way ANOVA followed by a Dunnett's multiple comparison test (confidence level 95%), referring to the control group (4 hpi): *p < 0.0332, **p < 0.0021, ***p < 0.0002, and ****p < 0.00001.

## Conflict of Interest

The authors declare no conflict of interest.

## Supporting information



Supporting Information

## Data Availability

The data that support the findings of this study are openly available in ChemRxiv at https://doi.org/10.26434/chemrxiv‐2024‐8b0gb, reference number 25732293.
