# Supplementary material for: Polysialosides Outperform Sulfated Analogs for Binding with SARS‐CoV‐2
Source: Small. 2025 Jul 16;21(34):2500719. doi: 10.1002/smll.202500719 (PMC12393028; doi:10.1002/smll.202500719)
Supplement: Supplementary file 1 — Supporting Information [file SMLL-21-2500719-s001.pdf]

NANO · MICRO
small

Supporting Information

for *Small*, DOI 10.1002/smll.202500719

Polysialosides Outperform Sulfated Analogs for Binding with SARS-CoV-2

Vinod Khatri, Nico Boback, Hassan Abdelwahab, Daniela Niemeyer, Tahlia M. Palmer, Anil Kumar Sahoo, Yannic Kerkhoff, Kai Ludwig, Julian Heinze, Dilara Balci, Jakob Trimpert, Rainer Haag, Tatyana L. Povolotsky, Roland R. Netz, Christian Drosten, Daniel C. Lauster* and Sumati Bhatia**

Polysialosides Outperform Sulfated Analogs for Binding with SARS-CoV-2

Vinod Khatri,[1,2,§] Nico Boback,[1,4,10,§] Hassan Abdelwahab,[6] Daniela Niemeyer,[5] Tahlia M. Palmer,[4,10] Anil Kumar Sahoo,[6*] Yannic Kerkhoff,[7,8] Kai Ludwig,[7] Julian Heinze,[5] Dilara Balci,[1] Jakob Trimpert,[9] Rainer Haag,[1] Tatyana L. Povolotsky,[10] Roland R. Netz,[6] Christian Drosten,[5] Daniel C. Lauster,[4*] Sumati Bhatia[3*]

[1]Freie Universität Berlin, Institute for Chemistry and Biochemistry, Takustr. 3, Berlin 14195 Germany

[2]Department of Chemistry, TDL Govt. College for Women Murthal, Sonipat-131027, Haryana, India

[3]Department of Chemistry, Faculty of Science and Engineering, Swansea University, Singleton Campus, Swansea SA2 8PP, United Kingdom

[4]Freie Universität Berlin, Institute of Pharmacy, Biopharmaceuticals, Kelchstr. 31, 12169 Berlin

[5]Institute of Virology, Campus Berlin Mitte, Charité – Universitätsmedizin Berlin, Charitéplatz 1, 10117 Berlin, Germany

[6] Freie Universität Berlin, Department of Physics, Arnimallee 14, 14195 Berlin, Germany

[7] Freie Universität Berlin, Forschungszentrum für Elektronenmikroskopie, Core-Facility BioSupraMol, Institute of Chemistry and Biochemistry, Fabeckstraße 36a, 14195 Berlin, Germany

[8] IT & Data Services, Zuse-Institut Berlin, Takustraße 7, 14195 Berlin, Germany

[9] Department of Pathobiology and Diagnostic Medicine, Kansas State University, 1800 Denison Avenue, Manhattan, KS, 66506, USA

[10]Freie Universität Berlin, SupraFAB, Institute for Chemistry and Biochemistry, Altensteinstr. 23a, 14195 Berlin, Germany

[§]Contributed equally

*Corresponding authors:

Sumati Bhatia, Email: sumati.bhatia@swansea.ac.uk

Daniel C. Lauster, Email: daniel.lauster@fu-berlin.de

Anil Kumar Sahoo, Email: aksahoo@zedat.fu-berlin.de

Table of contents

1. Experimental Section 3

1.1. Materials 3
1.2. Synthesis of aromatically modified sialic acid 3
1.3. Synthesis of dPG_{100} and dPG_{500} 5
1.4. Functionalisation of dPG_{100} and dPG_{500} 6
1.5. Synthesis of $dPG_{500}SA_{0.25}$, $dPG_{500}SA_{0.55}$ and $dPG_{100}SA_{0.20}$ 6
1.6. Synthesis of $dPG_{100}(SA_{aryl})_{0.20}$ 7
1.7. Synthesis of $dPG_{500}SA_{0.20}S_{0.20}$ 8
1.8 Synthesis of $dPG_{500}C_{0.20}$ 9
1.9 Synthesis of $dPG_{500}S_{0.25}$ and $dPG_{500}S_{0.55}$ 9
1.10 IR, NMR, HRMS, elemental and Zeta potential analysis 10
1.11 GPC analysis of dPG_{500} and dPG_{100} 11
1.12 Cryo-Transmission Electron Microscopy (cryo-TEM) and cryo- electron tomography (cryo-ET) measurements 11
1.13 Machine Learning-based Cryo-TEM segmentation 12

2. Supporting Figures and Tables 14-27

2.1 NMR Spectra 14-21
2.2 DLS Plots 21-22
2.3 Ensemble Docking Studies 22-23
2.4 MD Simulation and Ensemble Docking Data Analysis 23-27
2.5 Cell Viability 27
2.6 XPS plot 28

1. Experimental Section

1.1. Materials

Solvents and reagents of analytical quality are procured from commercial suppliers and utilized without further purification. Merck silica gel 60 F254 pre-coated thin layer chromatography (TLC) plates are utilized for monitoring the progress of reactions in the case of small molecules. Staining in 5% H_2SO_4 in ethanol and ceric solution is used to visualize the spots on TLC. The purification of small molecules was done using a column having silica gel with a mesh size of 100-200. Polymers were purified through dialysis against water by using a benzoylated membrane of MW cut-off 2000 Da.

1.2. Synthesis of aromatically modified sialic acid

Esterification

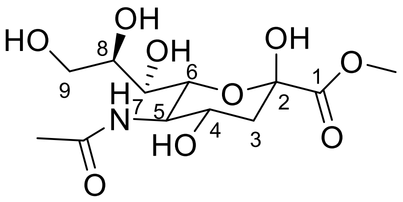

2

N-Acetylneuraminic acid (10.00 g, 32.33 mmol, 1.00 eq.) was tried to dissolve in dry methanol (200 mL) under argon atmosphere. After the suspension was stirred for 10 min., trifluoroacetic acid (TFA) (15.00 mL, 196.02 mmol, 6.06 eq.) was added dropwise. The reaction mixture was stirred at room temperature for 48 h. The suspension turned into a solution and the reaction progress was checked with TLC (30:70 MeOH:$CHCl_3$). After completion, the reaction mixture was filtered through a pad of celite, and the filtrate was evaporated under reduced pressure to obtain the desired compound as white solid with 98% yield. ^{1}H NMR (600 MHz, CD_3OD): δ 1.85 (dd, 1H, J = 13.0 and 11.4 Hz, H-3), 1.98 (s, 3H, CH_3 (NAc)), 2.18 (dd, 1H, J = 12.9 and 4.9 Hz, H3'), 3.44 (dd, 1H, J = 9.2 and 1.5 Hz, H-7), 3.58 (dd, 1H, J = 11.4 and 5.7 Hz, H-9), 3.66 (ddd, 1H, J = 8.9, 5.7 and 2.9 Hz, H-8), 3.74 (s, 3H, CH_3 ($COOCH_3$)), 3.76 (dd, 1H, J = 8.5 and 2.8 Hz, H-9'), 3.78 (t, 1H, J = 10.3 Hz, H-5), 3.96 (dd, 1H, J = 10.5 and 1.5 Hz, H-6), 4.00 (ddd, 1H, J = 11.4, 10.0 and 4.9 Hz, H-4); ^{13}C NMR (150 MHz, CD_3OD): δ 22.6, 40.7, 53.2, 54.3, 63.8, 67.9, 70.2, 71.6, 72.1, 96.7, 171.8, 175.2. HRMS (ESI) m/z calculated for $C_{12}H_{21}NO_9$: 346.1109 [M + Na^+], found 346.1181.

Acetylation and Chlorination

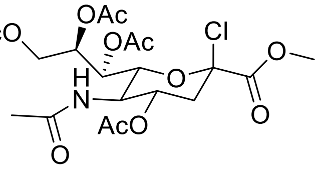

3

Compound **2** (5.00 g, 15.47 mmol, 1.00 eq.) was dissolved in acetyl chloride (AcCl) (150.00 mL, 2.10 mol, 135.85 eq.). After the reaction time of 16 h at room temperature, the acetyl chloride was removed under reduced pressure. The yellow viscous oil residue thus obtained was used without any further purification for the next step.

Aryl Substitution

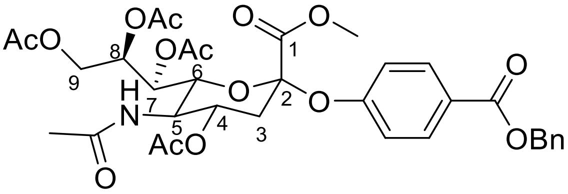

4

The impure compound **3** (9.55 g, 18.76 mmol, 1.00 eq.) was dissolved in DCM (150 mL). Benzyl 4-hydroxybenzoate (10.60 g, 46.44 mmol, 2.48 eq.) and TBAHS (5.77 g, 16.99 mmol, 0.91 eq.) were added. The yellow solution thus obtained was cooled down to 0 °C before 1 M aqueous NaOH solution (150 mL) was slowly added. After 30 min. of stirring at 0 °C, the ice bath was removed and the reaction mixture was stirred further for 48 h at room temperature. The reaction progress was monitored with TLC and on completion of the reaction; the compound was extracted with chloroform (2 x 50 mL). The combined organic layer was washed with water (1 x 100 mL), dried over sodium sulfate, and filtered through a pad of celite. The filtrate was concentrated under reduced pressure to obtain the crude product. The crude product thus obtained was purified with a silica column using 50% ethyl acetate in hexane followed by pure chloroform and later to a mixture of 2% methanol in chloroform as eluent. Thus the pure product **4** (4.30 g) was obtained as a white solid in 62.5% yield. ^{1}H NMR (600 MHz, $CDCl_3$): δ 1.92 (s, 3H, CH_3 (NAc)), 2.02 (s, 3H, CH_3 (OAc)), 2.04 (s, 3H, CH_3 (OAc)), 2.11 (s, 3H, CH_3 (OAc)), 2.16 (s, 3H, CH_3 (OAc)), 2.26 (t, 1H, J = 12.6 Hz, H-3), 2.70 (dd, 1H, J = 13.1 and 4.6 Hz, H-3'), 3.63 (s, 3H, CH_3 ($COOCH_3$)), 4.07-4.16 (m, 2H, H-5, H-9), 4.25 (dd, 1H, J = 12.5 and 1.9 Hz, H-9'), 4.55 (d, 1H, J = 10.8 Hz, H-6)), 4.96 (td, 1H, J = 11.9 and 4.7 Hz, H-4), 5.34 (s, 2H, H-7), 5.35 (dd, 1H, J = 8.0 and 1.6 Hz, H-7), 5.37 (dd, 1H, J =

4.9 and 2.3 Hz, H-8), 7.08 (d, 2H, J = 8.5 Hz), 7.34 (d, 1H, J = 6.7 Hz), 7.38 (t, 2H, J = 7.2 Hz), 7.44 (d, 2H, J = 7.0 Hz, 8.00 (d, 2H, J = 8.5 Hz; ^{13}C NMR (150 MHz, CDCl3): δ 20.9, 21.0, 21.1, 23.4, 38.6, 49.5, 53.3, 62.2, 66.7, 67.5, 68.6, 69.2, 73.9, 99.7, 118.6, 125.5, 128.4, 128.7 , 131.7, 136.3, 158.0, 166.1, 168.4 , 170.2 (2 peaks), 170.4, 170.7, 171.1. HRMS (ESI) m/z calculated for $C_{34}H_{39}NO_{15}$: 724.2212 [M + Na$^+$], found 724.2062.

Hydrogenation

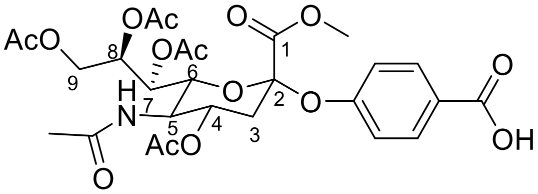

5

To a solution of compound **4** (4.30 g, 6.13 mmol, 1.00 eq.) in dry THF (70 mL), 10% Pd/C (0.43 g, 0.10 eq.) was added. The reaction mixture was stirred for 1 h under hydrogen atmosphere. The reaction progress was checked with TLC. After completion, 10% Pd/C was removed by filtration through silica gel with a mixture of methanol:THF (10:90) as the solvent. The solvent was evaporated under reduced pressure to obtain the pure desired product **5** as a white solid (3.72 g) in quantitative yield. ^{1}H NMR (600 MHz, $CDCl_3$): δ 1.92 (s, 3H, CH_3 (NAc)), 2.04 (s, 6H, CH_3 (OAc)), 2.11 (s, 3H, CH_3 (OAc)), 2.16 (s, 3H, CH_3 (OAc)), 2.28 (t, 1H, J = 12.6 Hz, H-3), 2.70 (dd, 1H, J = 13.0 and 4.6 Hz, H-3'), 3.63 (s, 3H, CH_3 ($COOCH_3$)), 4.10-4.17 (m, 2H, H-9), 4.25 (dd, 1H, J = 12.5 and 2.5 Hz, H-9'), 4.57 (dd, 1H, J = 10.8 and 1.9 Hz, H-6), 4.96 (ddd, 1H, J = 12.2, 10.3 and 4.6 Hz, H-4), 5.37 (d, 1H, J = 1.9 Hz, H-7), 5.39 (dd, 1H, J = 5.1 and 2.5 Hz, H-8), 7.10 (d, 2H, J = 9.0 Hz), 8.03 (d, 2H, J = 9.0 Hz); ^{13}C-NMR (150 MHz, CDCl3): δ 20.9, 21.0, 21.1, 23.4, 38.7, 49.5, 53.3, 62.2, 67.4, 68.6, 69.2, 73.8, 99.7, 118.6, 128.4, 132.2, 158.5, 168.4, 170.2, 170.4, 170.6, 170.8, 171.1. HRMS (ESI) m/z calculated for $C_{27}H_{33}NO_{15}$: 634.1742 [M + Na$^+$], found 634.1743. FT-IR (Film, cm^{-1}): 3264, 3064, 2937, 2878, 1742, 1717, 1686, 1660, 1210, 1168, 1122, 1066, 1032, 772.

1.3. Synthesis of dPG$_{100}$ and dPG$_{500}$

dPG$_{100}$ and dPG$_{500}$ were synthesized using a procedure as reported by Imran ul-haq et.al[1] and characterized by NMR, GPC, and MALLS.

dPG$_{500}$ GPC (DMF): M_n = 481 kDa, M_w = 552 kDa, PDI = 1.15

GPC-MALLS (H_2O): M_n= 436 kDa, M_w = 481 kDa, PDI =1.10

dPG$_{100}$ GPC (H_2O): M_n = 16366 Da, M_w = 50038 Da, PDI = 3.05

1.4. Functionalisation of dPG$_{100}$ and dPG$_{500}$

dPG$_{100}$-N_3 and dPG$_{500}$-N_3 were synthesized by mesylation followed by azidation of dPG$_{100}$-OMs and dPG$_{500}$-OMs as reported by literature procedure.[2] Further, dPG$_{100}$-NH_2 was synthesized by reducing the dPG$_{100}$-N_3 using PPh_3 in THF-H_2O following the previously reported procedure.[3]

1.5. Synthesis of dPG$_{500}$SA$_{0.25}$, dPG$_{500}$SA$_{0.55}$ and dPG$_{100}$SA$_{0.20}$

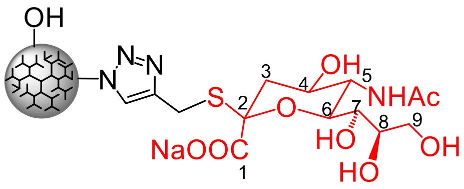

dPG$_{500}$SA$_{0.25}$
dPG$_{500}$SA$_{0.55}$
dPG$_{100}$SA$_{0.20}$

dPGN$_3$ with DF = 0.25 (200.00 mg, 0.50 mmol N_3 groups) was dissolved in 10 mL of DMF solvent followed by addition of a solution of prop-2-ynyl-α-thiosialoside (354 mg, 0.65 mmol, 1.30 eq. with respect to N_3 groups) in DMF (1.00 mL). The $CuSO_4.5H_2O$ (24.90 mg, 0.10 mmol, 0.20 eq. with respect to N_3 groups) was dissolved in 0.20 mL of H_2O and added to the solution of sodium ascorbate (198.00 mg, 1.00 mmol, 2.00 eq. with respect to N_3 groups) in 0.20 mL H_2O. The resulting solution was added dropwise to the solution containing dPGN$_3$ and prop-2-ynyl-α-thiosialoside. The reaction mixture was degassed thoroughly with argon for 5 minutes and then allowed to stir for 3 days at 50 °C temperature under argon atmosphere. The progress of the reaction was monitored by IR. When the peak at ~2100 cm^{-1} corresponding to azide stretching frequency completely disappeared, the solvent was evaporated at the rotary evaporator. 2 M NaOH (5 mL) was added to the residue and stirred at room temperature for 2 hours. The resulting reaction mixture thus obtained was neutralized with 1 N HCl and was dialyzed first against water and aqueous EDTA prepared with 2M NaOH solution for 2 days and again using only water for 4 days. The solvent of the dialysis was changed thrice a day. The aqueous solution obtained after dialysis was lyophilized to afford the pure dPGSA.

dPG$_{500}$SA$_{0.25}$

It was obtained in 64% yield. ^{1}H NMR (600 MHz, D_2O): δ 1.27 (brs, 1H, H-3a), 2.02 (s, 3H, CH_3 (NHAc)), 2.72-2.80 (m, 1H, H-3e), 3.61-4.58 [m, SA (H-4, H-5, H-6, H-7, H-8, H-9, NH), SCH_2, dPG scaffold], 7.93 (s, 1H, C=CH), IR (thin film, cm^{-1}): 3293, 2924, 2876, 1607, 1553,

1431, 1370, 1317, 1283, 1262, 1109, 1055, 953. The given ^{1}HNMR spectrum of dPG$_{500}$-SA$_{0.25}$ shows DF = 0.27, M$_w$ (NMR analysis) ~ 1247.78 kDa, CHNS analysis (%): 9.04 (N), 64.65 (C), 4.83 (S), 2.39 (H).

dPG$_{500}$SA$_{0.55}$

It was obtained in 68% yield. ^{1}H NMR (600 MHz, D_2O): δ 1.68-1.84 (m, 1H, H-3a), 2.02 (s, 3H, CH_3 (NHAc)), 2.72-2.87 (m, 1H, H-3e), 3.35-4.02 [m, SA (H-4, H-5, H-6, H-7, H-8, H-9, NH), SCH_2, dPG scaffold], 7.93 (brs, 1H, C=CH); FT-IR (thin film, cm^{-1}): 3297, 2920, 2873, 1613, 1550, 1431, 1372, 1317, 1109, 1065, 942. The given ^{1}HNMR spectrum of dPG$_{500}$-SA$_{0.50}$ shows DF = 0.54, M$_w$ (NMR analysis) ~ 1995.62 kDa, CHNS analysis (%): 11.63 (N), 61.83 (C), 6.63 (S), 2.44 (H).

dPG$_{100}$SA$_{0.20}$

It was obtained in 51% yield. ^{1}H NMR (600 MHz, D_2O): δ 1.76-1.83 (m, 1H, H-3a), 2.02 (s, 3H, CH_3 (NHAc)), 2.77-2.84 (m, 1H, H-3e), 3.64-4.59 [m, SA (H-4, H-5, H-6, H-7, H-8, NH), SCH_2, dPG scaffold), 7.96 (brs, 1H, C=CH); FT-IR (thin film, cm^{-1}): 3277, 2918, 2873, 1603, 1551, 1458, 1368, 1321, 1283, 1250, 1112, 1058, 955. The given ^{1}HNMR spectrum of dPG$_{100}$-SA$_{0.20}$ shows DF = 0.22 M$_w$ (NMR analysis) ~ 221.74 kDa.

1.6. Synthesis of dPG$_{100}$(SA$_{aryl}$)$_{0.20}$

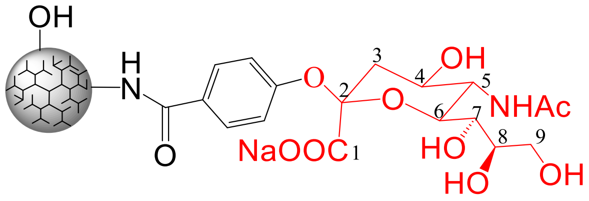

dPG$_{100}$(SA$_{aryl}$)$_{0.20}$

Compound SA$_{aryl}$ (0.63 g, 1.03 mmol) was dried over vacuum and dissolved in dry DMF. To this solution, EDC HCl (0.19 g, 1.01), HOBt (0.16 g, 1.19 mmol), and DIPEA (0.39 mL, 2.11 mmol) were added and allowed to stir for 30 minutes. After 30 minutes, dPG-NH_2 (0.25 g, 0.74 mmol, solution in DMF) was added and the reaction mixture was allowed to stir for 24 h at 45 °C. Reaction mixture was quenched by adding water. The solvent was evaporated on the rotatory evaporator. After this 2 M NaOH (10 mL) was added and stirred for 2 h followed by dialysis first against water and methanol mixture for 1 day and again using only water for 4 days. The solvent of the dialysis was changed thrice a day. The aqueous solution obtained after dialysis was lyophilized to afford the pure dPG$_{100}$(SA$_{aryl}$)$_{0.20}$ with a quantitative conversion.

The degree of functionalization was confirmed by the ^{1}H-NMR spectrum of the pure product, by correlating the CH_3-Ms peak at 2.04 ppm with the polyglycerol backbone protons. The product was obtained with 65% yield. ^{1}H-NMR (600 MHz, D_2O): δ 1.94-1.96 (m, 1H, H-3a), 2.05 (s, 3H, CH_3, NHAc), 2.86-2.89 (m, 1H, H-3e), 3.54-3.99 [m, SA (H-4, H-5, H-6, H-7, H-8, H-9, NH), SCH_2, dPG scaffold], 6.91 (brs, 2H, Ar), 7.23 (brs, 1H, CH=C), 7.70 (brs, 2H, Ar), IR (Film, cm^{-1}): 3317, 3117, 2919, 2880, 1625, 1611, 1232, 1067, 768. The given ^{1}HNMR spectrum of $dPG_{100}(SA_{aryl})_{0.20}$ shows DF = 0.22, M_w (NMR analysis) ~ 228.27 kDa.

1.7. Synthesis of $dPG_{500}SA_{0.20}S_{0.20}$

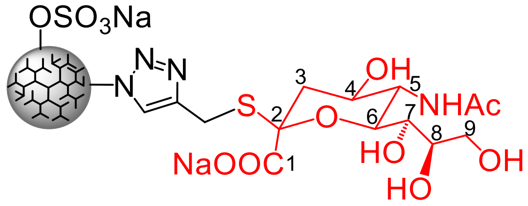

$dPG_{500}SA_{0.20}S_{0.20}$

$dPG_{500}SAprotected_{0.20}$ (0.63 g, 0.67 mmol, 1.00 eq.) was dissolved in 20 mL dry DMF. The solvent was evaporated at reduced pressure and this process was repeated two times. After that, 20 mL dry DMF was added together with SO_3/Pyridine (0.118 g, 0.74 mmol, 1.1 eq., with respect to OH group) dropwise to the mixture. The resulting reaction mixture was stirred overnight at 70 °C. Subsequently, DMF was evaporated at reduced pressure. Further, 1 M NaOH (1 x 30 mL) solution was added to the residue and basified to pH 14. The resulting mixture was dialyzed in EDTA for two days and then in water for next two days to obtain the pure product $dPG_{500}SA_{0.20}S_{0.20}$. ^{1}H NMR (700 MHz, D_2O): δ 1.84 (brs, 1H, H-3a), 2.05 (s, 3H, CH_3, NHAc), 2.81 (brs, 1H, H-3e), 3.58-4.60 [m, SA (H-4, H-5, H-6, H-7, H-8, H-9, NH), SCH_2 and dPG scaffold], 7.97 (brs, 1H, C=CH). IR (Film, cm^{-1}): 3310, 2920, 2882, 1642, 1382, 1222, 1053, 800. The given ^{1}H NMR spectrum of dPG_{500}-$SA_{0.20}S_{0.20}$ shows DF = 0.18 with respect to sialic acid and M_W (NMR analysis) ~ 1468.68 kDa. CHNS analysis (%): 6.66 (N), 41.17 (C), 6.13 (S), 7.03 (H).

1.8 Synthesis of $dPG_{500}C_{0.20}$

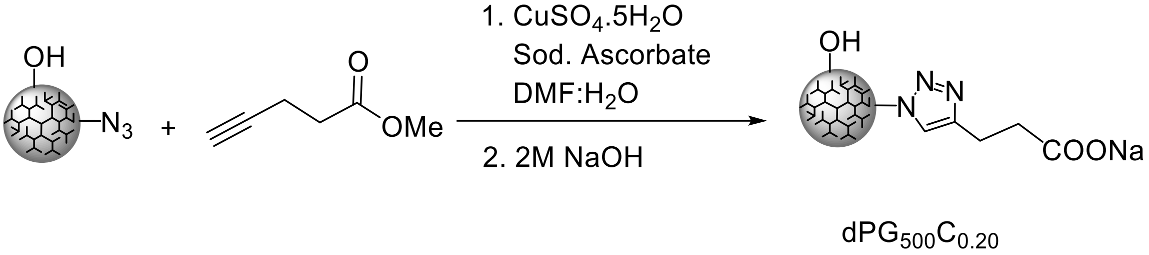

Scheme S1: Chemical structure of the $dPG_{500}C_{0.20}$ conjugate.

dPG-N_3 (200.00 mg, 0.54 mmol) with DF 20% and methyl pent-4-ynoate (66.50 mg, 0.59 mmol) was dissolved in DMF (10 mL). The $CuSO_4.5H_2O$ (26.90 mg, 0.11 mmol) solution was prepared by dissolving it in 0.20 mL of water and added to the solution of sodium ascorbate (213.00 mg, 1.08 mmol) in 0.40 mL of water. The resulting solution was added dropwise to the solution of the polymer. The reaction mixture was degassed thoroughly with argon on ultrasonication for 5 minutes and then allowed to stir at 50 °C for three days under argon atmosphere. The progress of the reaction was monitored by IR. When the peak at ~2100 cm^{-1} corresponding to azide stretching frequency completely disappeared, the solvent was evaporated at the rotary evaporator. After this 2 M NaOH solution (10 mL) was added to the reaction mixture and stirred overnight followed by neutralization with 1N HCl. The dialysis of reaction mixture was done first against methanol for 2 days and water : methanol (1:1) mixture for 1 day and only in water for 2 days. The solvent of the dialysis was changed thrice a day. The aqueous solution obtained after dialysis was lyophilized to afford the pure $dPG_{500}C_{0.20}$ with a 70% yield. ^{1}H NMR (500 MHz, H_2O):δ 2.62 (brs, 2H, -$CH_2C\mathbf{H_2}COONa$), 2.91 (brs, 2H, -$C\mathbf{H_2}CH_2COONa$), 3.59-4.48 (m, 22H, dPG scaffold), 7.82 (brs, 1H, C=C**H**); FT-IR (Film, cm^{-1}): 3640-2400, 1722, 1620, 1212, 1045. The given ^{1}HNMR spectrum of $dPG_{500}C_{0.20}$ shows DF = 0.22, M_w (NMR analysis) ~ 715.41 kDa.

1.9 Synthesis of $dPG_{500}S_{0.25}$ and $dPG_{500}S_{0.55}$

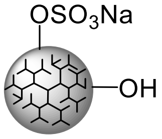

$dPG_{500}S_{0.25}$
$dPG_{500}S_{0.55}$

dPG_{500} (500 kDa, 0.11g, 0.00022 mmol, 1.00 eq.) was dissolved in 15 mL DMF. The solvent was removed in vacuum. Subsequently, dry DMF (2 x 15 mL) was added and then removed in vacuum. Further, 15 mL dry DMF was added together with SO_3/Pyridine (0.057 g, 0.36 mmol, 1.2 eq., with respect to OH group) drop-wise to the mixture. The resulting solution was stirred overnight. Subsequently, DMF was evaporated using a rotary evaporator. 1 M NaOH solution was added to the mixture and basified to pH 14. The residue was dialyzed in a saturated sodium chloride solution for one day followed by dialysis against water for 3 days. The solvent of dialysis was changed thrice a day. The product was determined by ^{1}H-NMR and IR spectroscopy. The degree of functionalization was determined by elemental analysis.

$dPG_{500}S_{0.25}$

It was obtained in quantitative yield. ^{1}H NMR (700 MHz, D_2O): δ 3.60-4.07 (m, CH_2 and CH, dPG scaffold). IR (thin film, cm^{-1}): 3373, 2868, 1650, 1457, 1328, 1254, 1059, 861. CHNS analysis: 0.03 (N), 90.25 (C), 4.32 (S), 7.32 (H). CHNS analysis (%): 0.03 (N), 90.25 (C), 4.32 (S), 7.32 (H). The given CHNS analysis of $dPG_{500}S_{0.25}$ shows DF = 0.24, M_w (CHNS analysis) ~ 665.12 kDa.

$dPG_{500}S_{0.55}$

It was obtained in quantitative yield. ^{1}H NMR (700 MHz, D_2O): δ 3.72-4.66 (m, CH_2 and CH, dPG scaffold). IR (thin film, cm^{-1}): 3364, 2876, 1635, 1465, 1246, 1064, 869. CHNS analysis (%): 0.02 (N), 70.32 (C), 10.03 (S), 5.87 (H). The given CHNS analysis of $dPG_{500}S_{0.55}$ shows DF = 0.55, M_w (CHNS analysis) ~ 878.60 kDa.

1.10 IR, NMR, HRMS, elemental and Zeta potential analysis

Infrared (IR) transmission spectra were recorded on Nicolet AVATAR 320 FT-IR 5 SXC with a DTGS detector from 650 to 4000 cm^{-1} (Thermo Fisher Scientific, Waltham, MA, USA). ^{1}H and ^{13}C NMR spectra were measured on the Bruker BioSpin 700 MHz and Jeol 600 MHz using solvent residual peak as internal standard, where the values of chemical shift are shown on δ scale and coupling constant (*J*) are in Hz. The ESI was measured using a TSQ 7000 (Finnigan Mat) instrument. The elemental analysis measurements were conducted on an analyzer VARIO EL (Elementar instrument). The zeta potential measurements were done using NanoDLS (Brookhaven Instruments Corp.) at 25 °C PB (10 mM, 7.4 pH) at 1 mg/mL particle concentration.

1.11 GPC analysis of dPG_{500} and dPG_{100}

The average molar masses of the dPG_{100} and dPG_{500} were determined using GPC analysis. The complete distribution (Mn, Mp, Mw, dispersity) was obtained with the connected refractive index detector (RI) operated at 50 °C. Besides the RI detector, a DAWN-EOS multi-angle laser light scattering (MALLS) (Wyatt Technology Inc., Santa Barbara CA) detector was used. The samples were measured under highly diluted conditions (5 mg/ml) with a GPC device build-up with an Agilent 1100 solvent-delivering system with manual and pump injection, an Agilent differential refractometer and three equipped 30 cm columns (PPS: Polymer Standards Service GmbH, Germany; Suprema 100 Å, 1000 Å, 3000 Å with 5 and 10 mm particle size), operated at r.t.. The separation of the aqueous polymer solution was run with water and 0.1 M $NaNO_3$ as the mobile phase with a flow rate of 1 ml/min. As a calibrant certified pullulan (linear) and dextran (branched) from PSS were used. The data analysis was done using WinGPC from PSS.

1.12 Cryo-Transmission Electron Microscopy (cryo-TEM) and cryo- electron tomography (cryo-ET) measurements

Perforated carbon film-covered microscopical 200 mesh grids (R1/4 batch of Quantifoil, MicroTools GmbH, Jena, Germany) were cleaned with chloroform and hydrophilised by 60 s glow discharging at 10 W in a CCU-010 device (safematic GmbH, Zizers, Switzerland) before 4 µL aliquots of the functionalized polyglycerol solutions (1 mg/mL in PBS) were applied to the grids. In the case of the virus binding test, the corresponding polyglycerol solution (1 mg/mL) was previously incubated with the same volume of SARS-CoV-2 virions in PBS for 30 min with gentle shaking at rt. The samples were vitrified by automatic blotting and plunge freezing with a FEI Vitrobot Mark IV (Thermo Fisher Scientific Inc., Waltham, Massachusetts, USA) using liquid ethane as cryogen. The vitrified specimens were transferred to the autoloader of a FEI TALOS ARCTICA electron microscope (Thermo Fisher Scientific Inc., Waltham, Massachusetts, USA). The microscope is equipped with a high-brightness field-emission gun (XFEG) operated at an acceleration voltage of 200 kV. Micrographs were acquired on a FEI Falcon 3 4k×4k direct electron detector (Thermo Fisher Scientific Inc., Waltham, Massachusetts, USA) using either a 100 µm objective aperture or a volta phase plate at a nominal magnification of 28,000 corresponding to a calibrated pixel size of 3.69 Å/pixel, respectively.

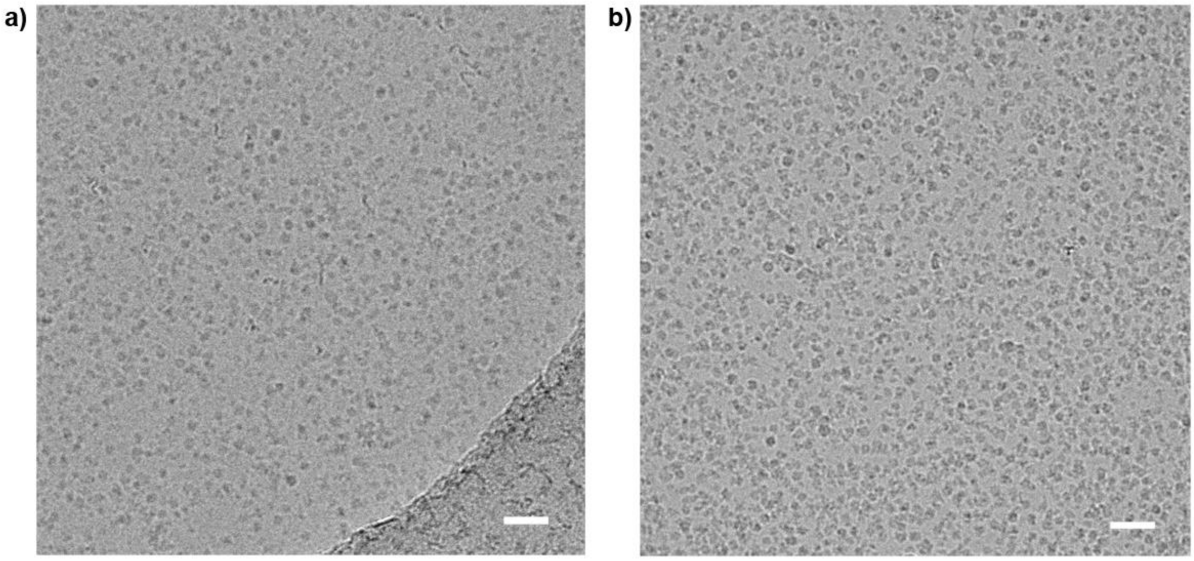

Figure S1: Cryo-TEM images of (a) dPG_{500} and (b) $dPG_{500}SA_{0.55}$, embedded in vitreous ice (concentration 1 mg/ml) Scale bar corresponds to 50 nm.

To obtain spatial information of the samples, tomograms were recorded on the TALOS® ARCTICA transmission electron microscope (ThermoFisher Scientific Inc., Waltham (MA), USA) at 200 kV. For this purpose, single axis tilt series (±64° in 2° tilt angle increments) were acquired with a FEI Falcon 3EC 4k×4k direct electron detector using a Volta Phase Plate at 28 K primary magnification with a total dose lower than 100 e-/Å2. Reconstruction of the tomograms was performed with binned data (binning factor 2) using ThermoFisher Inspect3D software, version 3.1.0. The increased contrast provided by the phase plate facilitated the alignment of the individual tilt images, but also slightly reduced the transmission of high spatial frequencies.

1.13 Machine Learning-based Cryo-TEM segmentation

To segment and visualize the interaction between sialoside-functionalized dPG nanoparticles and SARS-CoV-2 spike protein RBDs, a machine learning approach using the Trainable Weka Segmentation (TWS) Classifier in Fiji was implemented. The training architecture was based on a FastRandomForest algorithm with the following parameters: 200 decision trees, 2 randomly selected features per node, and a random seed value. Feature extraction included a combination of Gaussian blur, Sobel filter, Hessian matrix, difference of Gaussians, membrane projections, anisotropic diffusion, and neighbourhood analysis. The membrane thickness was set to 1 pixel, with a patch size of 19 pixels. The sigma range for Gaussian features extended from 1.0 to 16.0. Five distinct classes were defined for segmentation: background (bg), virus, spike proteins, dPG_{500} nanoparticles, and contamination. The classifier was trained with a batch

size of 100, utilizing 16 threads for parallel processing. The maximum tree depth was not limited (maxDepth = 0), and the number of decimal places for numerical precision was set to 2.

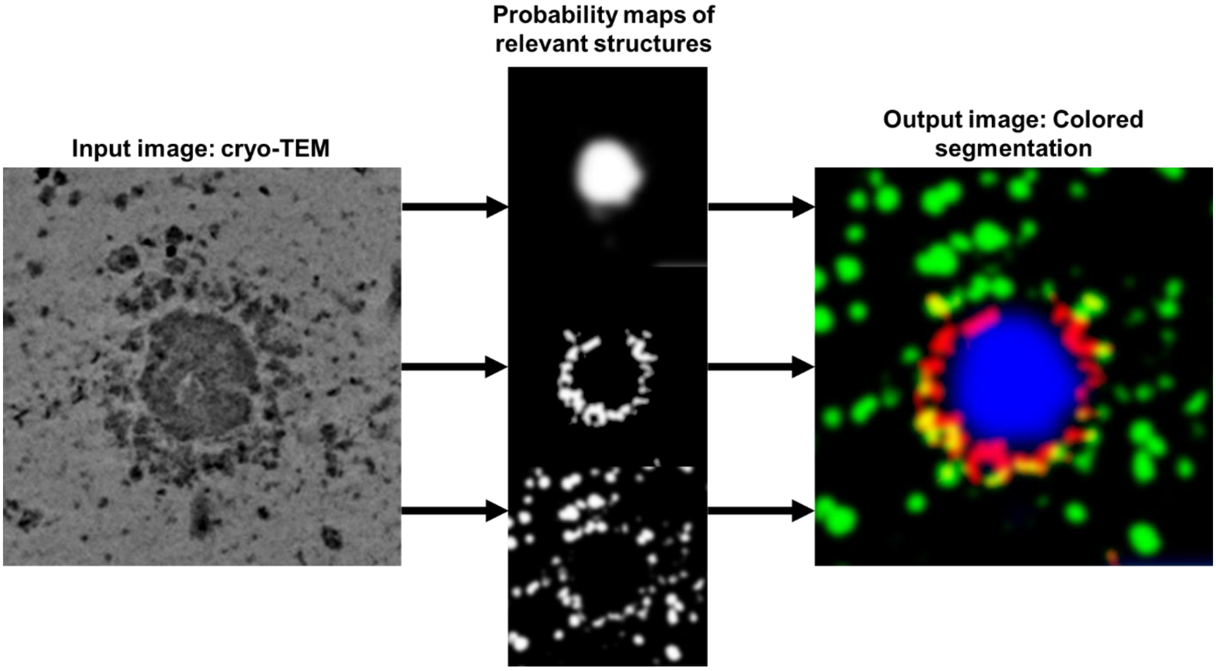

Figure S2: Illustration of the progression from raw data to the final segmented visualization.

The structures corresponding to the classes were manually annotated and the model was trained iteratively until the average loss per epoch fell below 0.01, ensuring accuracy and reliability of the segmentation. Following the training, the resulting pixel-based probability maps for the classes 'virus', 'spike proteins', and 'dPG_{500} nanoparticles' were color-coded (blue, red, and green, respectively). These probability maps were then smoothed using a Gaussian blur filter to produce the final colored segmentation, enhancing the visual representation of the different components while maintaining the integrity of the structural information.

2. Supporting Figures and Tables

2.1 NMR Spectra

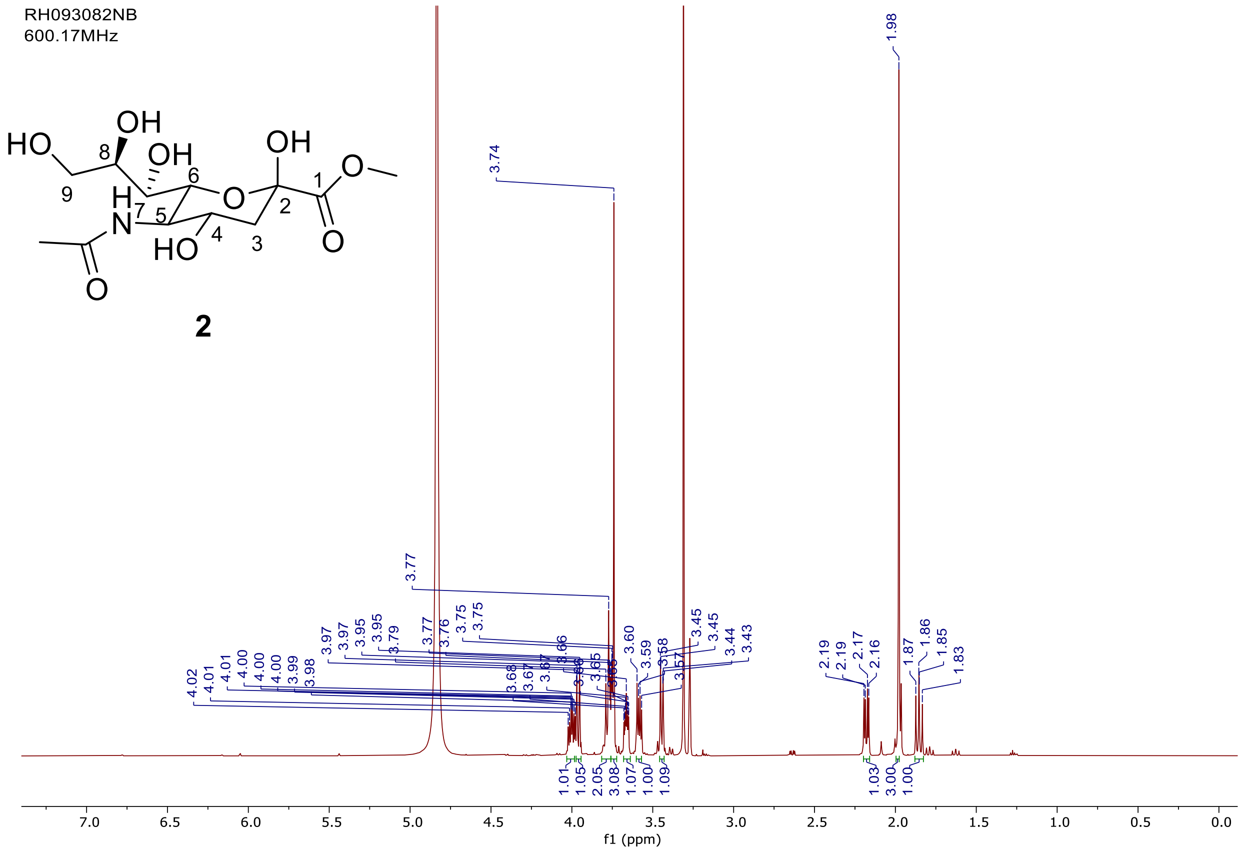

Figure S3: ^{1}H NMR spectrum (600 MHz, CD_3OD) of compound **2**.

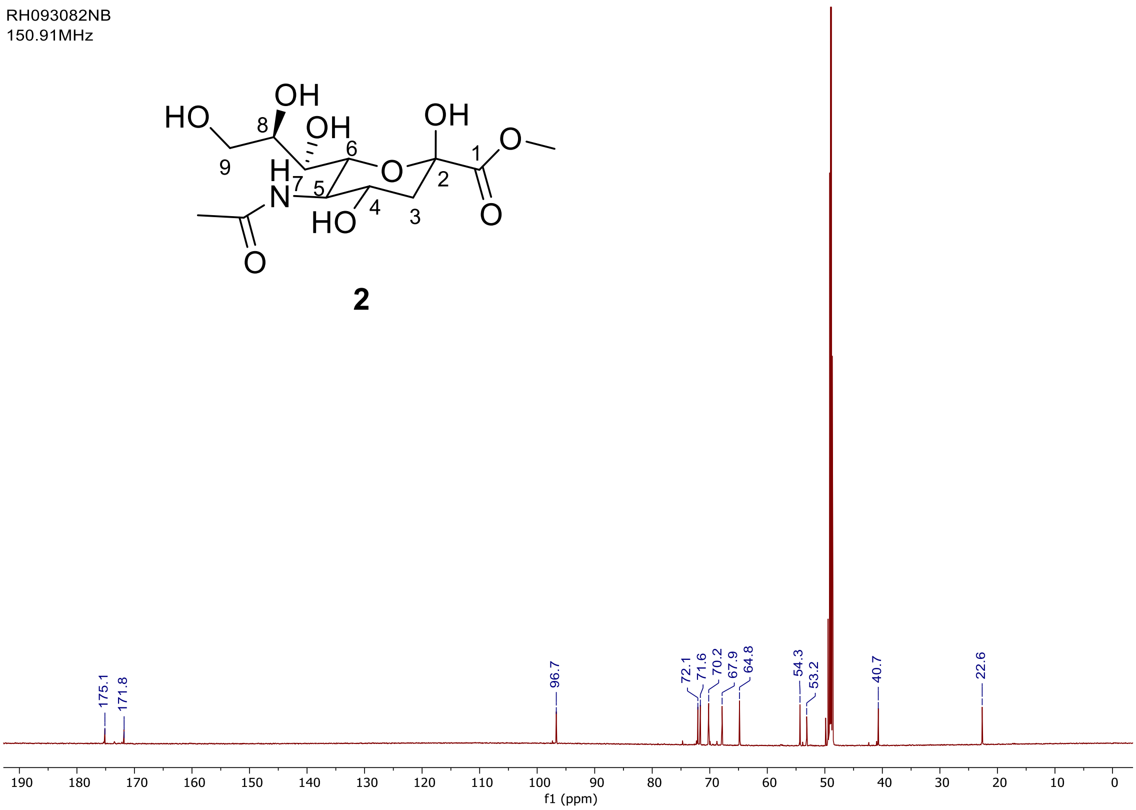

Figure S4: ^{13}C NMR spectrum (150.9 MHz, CD_3OD) of compound **2**.

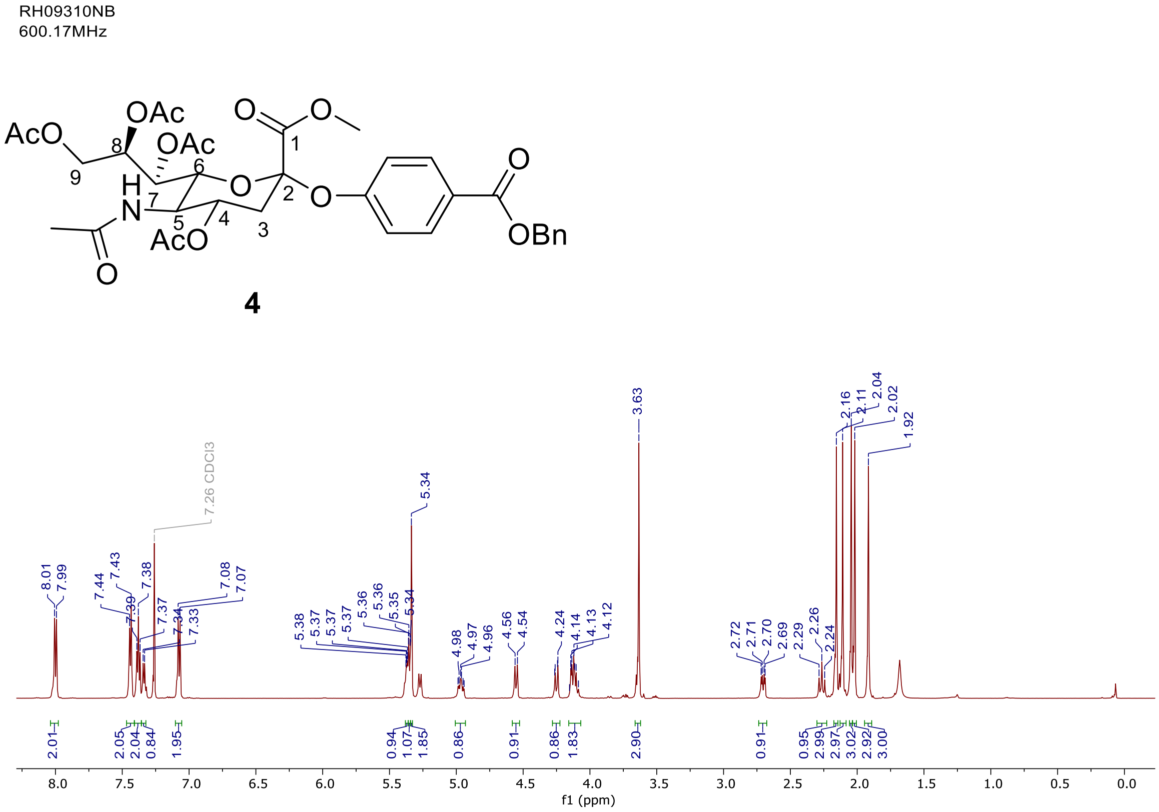

Figure S5: ^{1}H NMR spectrum (600 MHz, $CDCl_3$) of compound **4**.

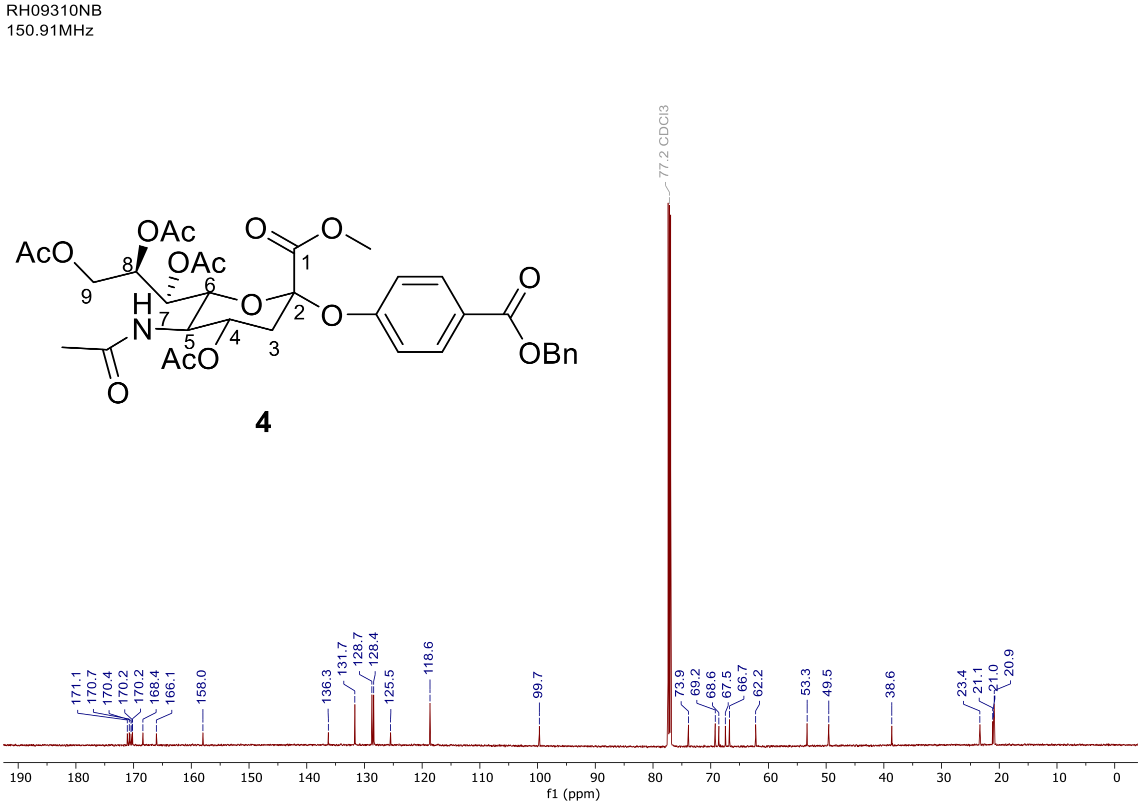

Figure S6: ^{13}C NMR spectrum (150.9 MHz, $CDCl_3$) of compound **4**.

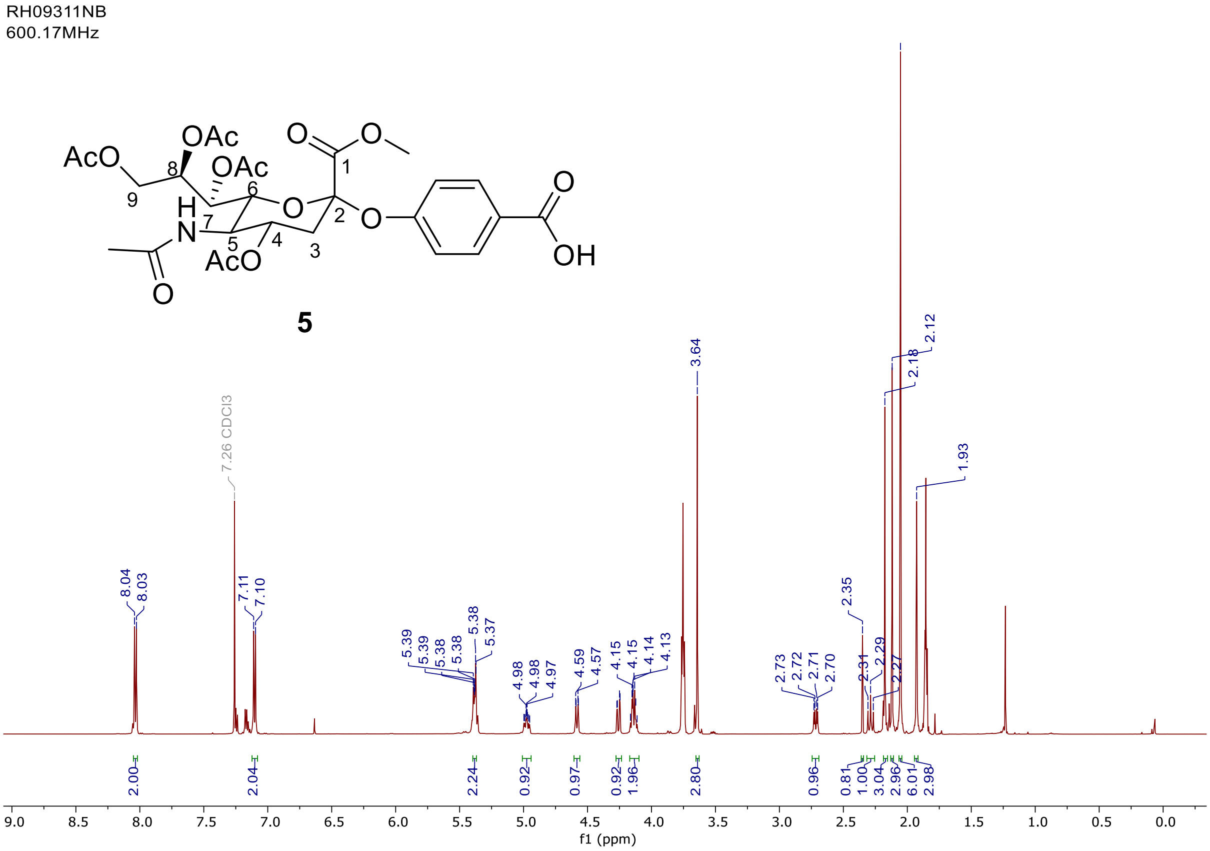

Figure S7: ^{1}H NMR spectrum (600 MHz, $CDCl_3$) of compound **5**.

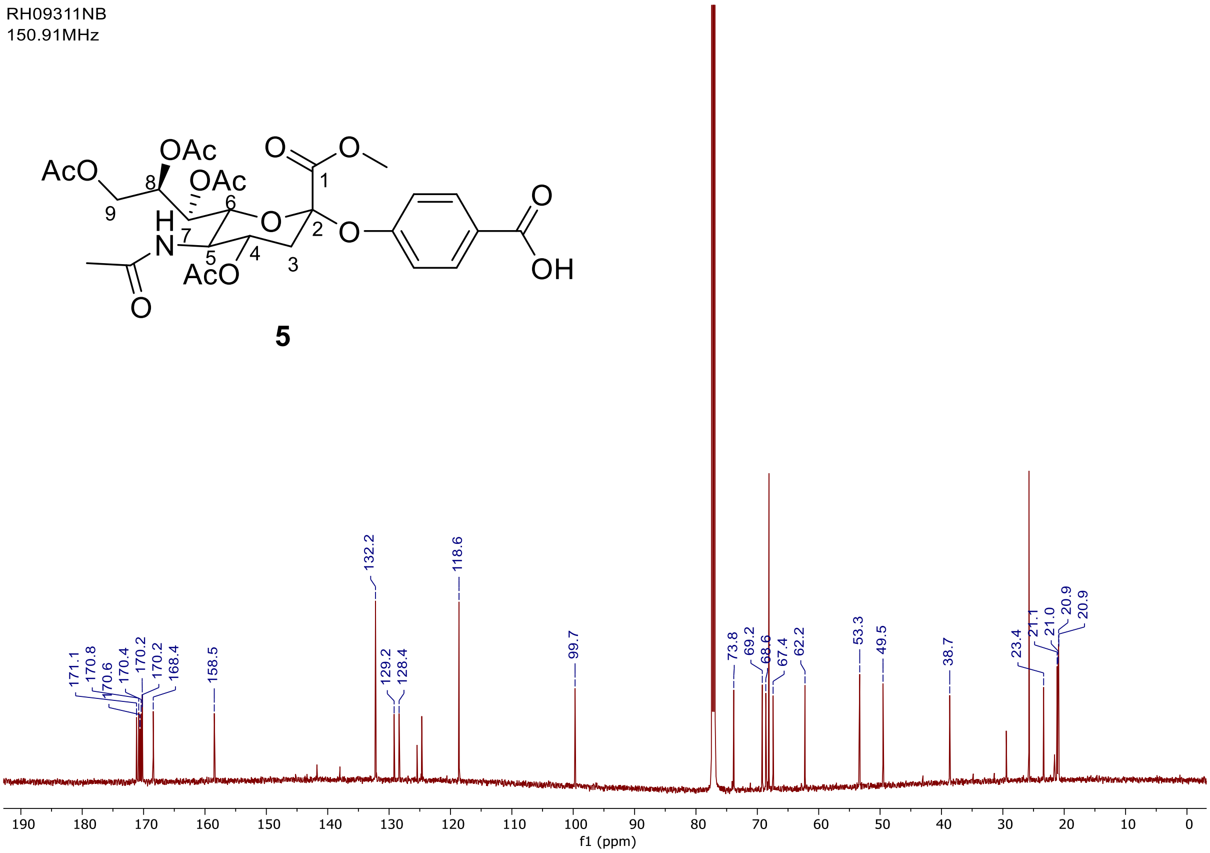

Figure S8: ^{13}C NMR spectrum (150.9 MHz, $CDCl_3$) of compound **5**.

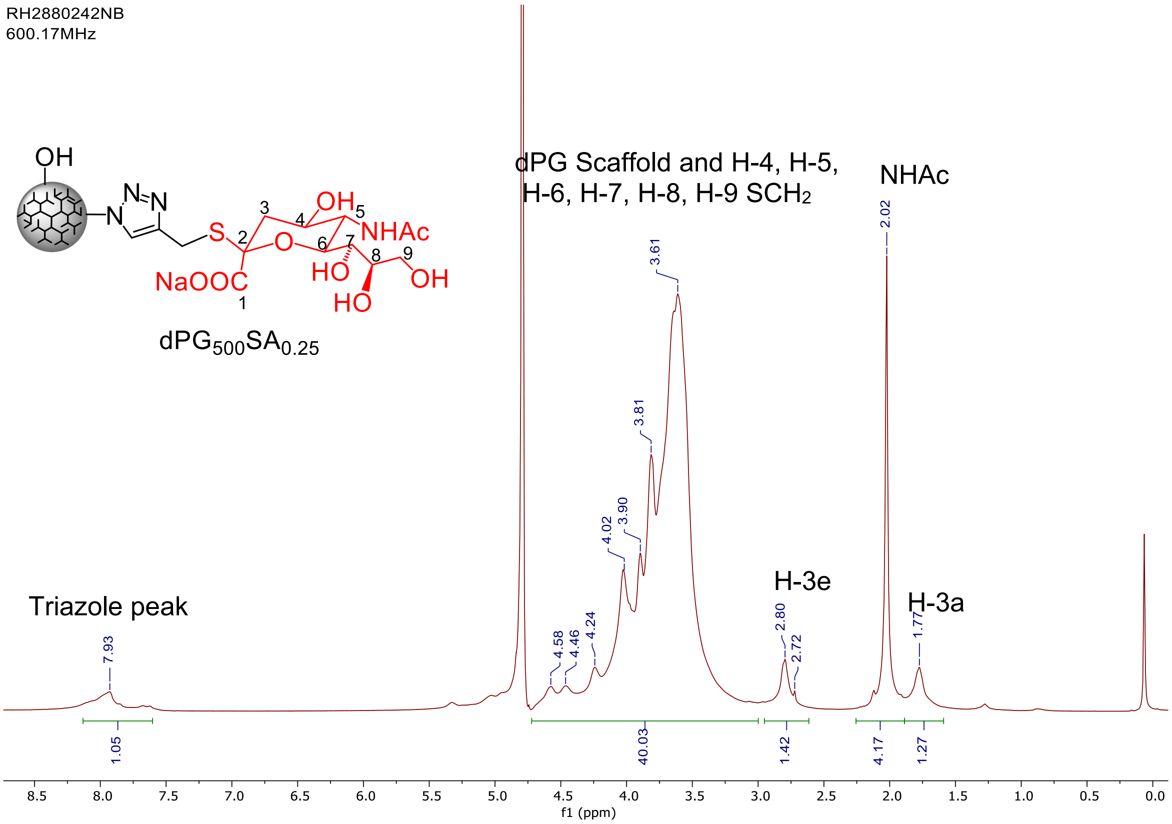

Figure S9: ^{1}H-NMR spectrum (600 MHz, D_2O) of dPG$_{500}$SA$_{0.25}$.

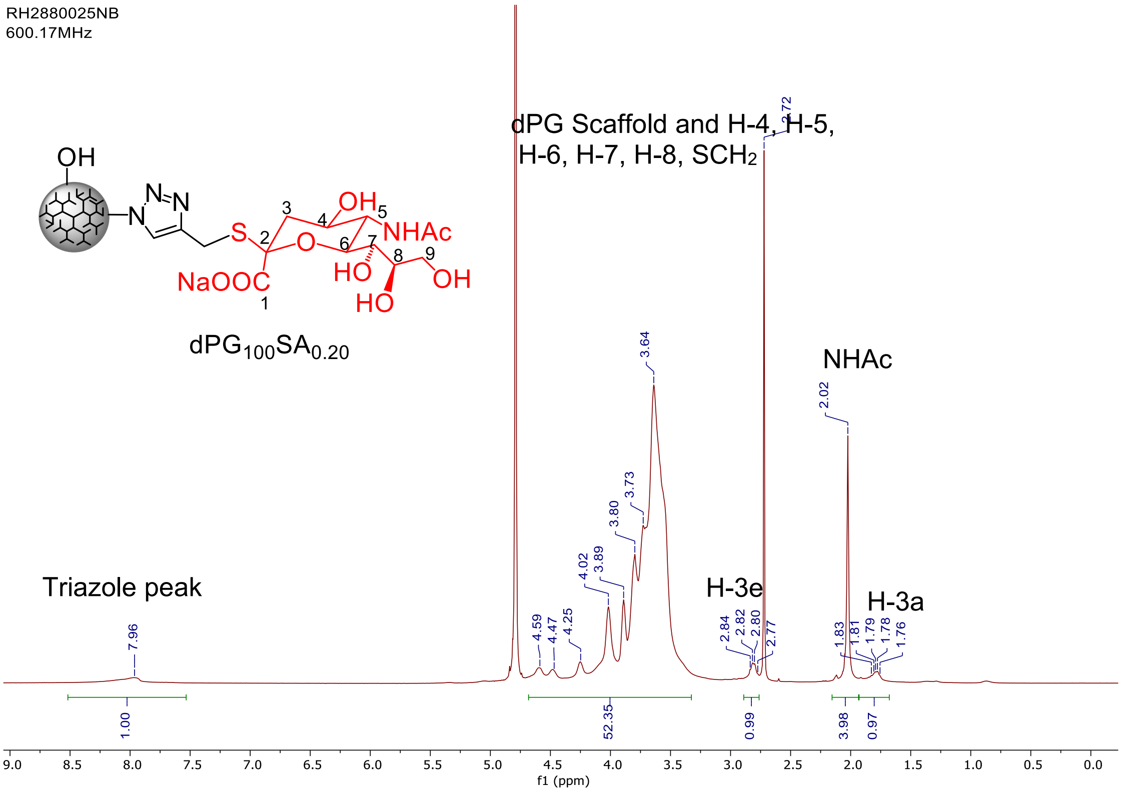

Figure S10: ^{1}H-NMR spectrum (600 MHz, D_2O) of $dPG_{100}SA_{0.20}$.

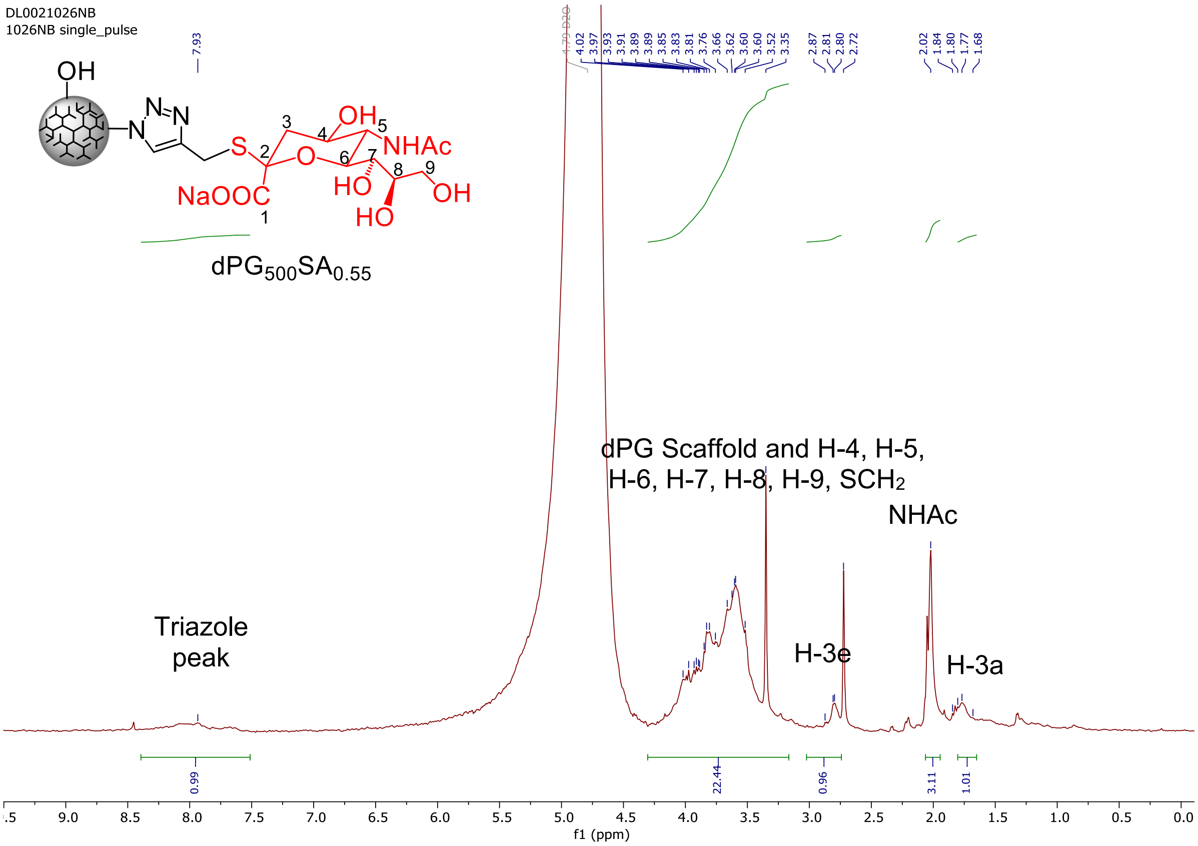

Figure S11: ^{1}H-NMR spectrum (600 MHz, D_2O) of $dPG_{500}SA_{0.55}$.

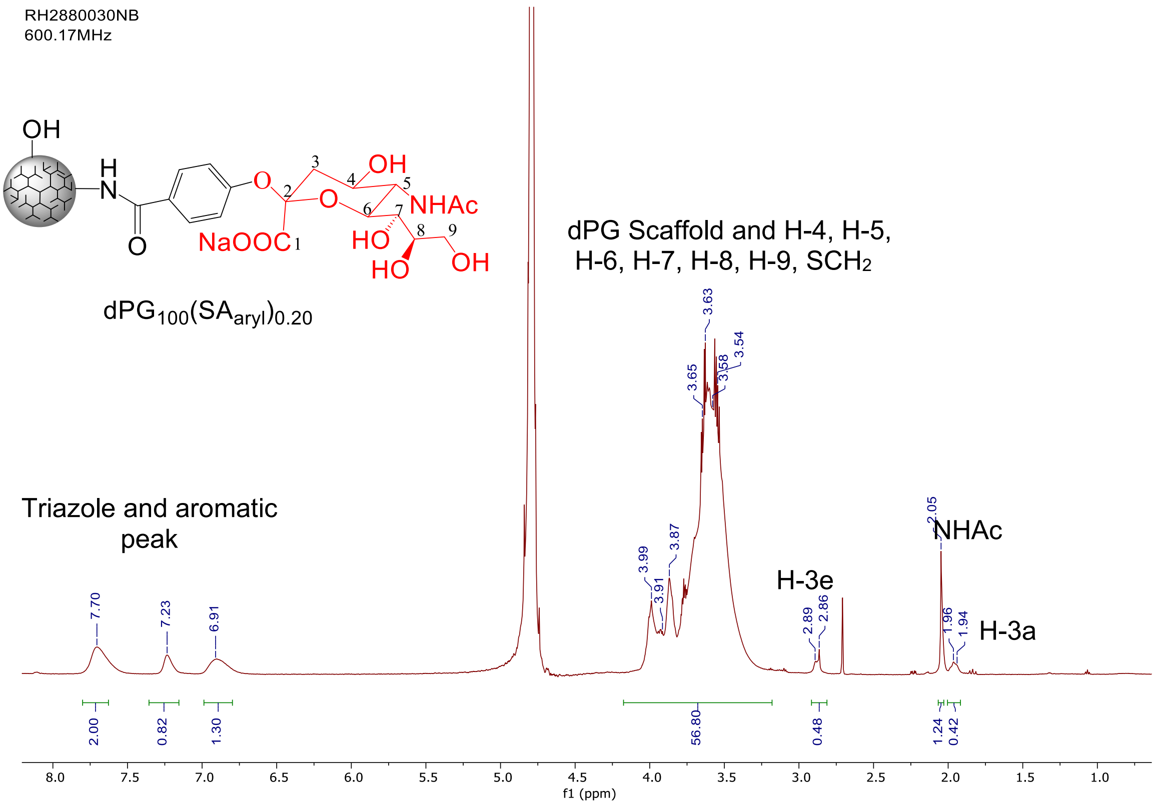

Figure S12: ^{1}H-NMR spectrum (600 MHz, D_2O) of dPG$_{100}$(SAaryl)$_{0.20}$.

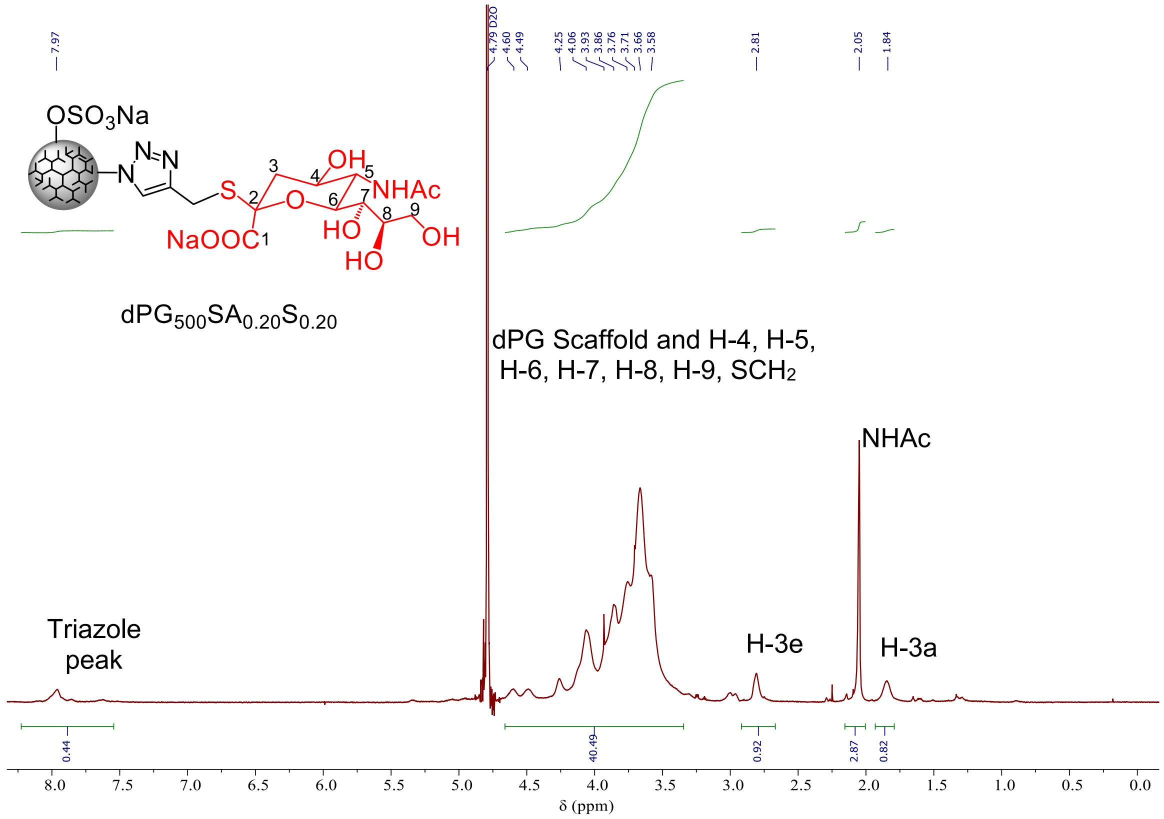

Figure S13: ^{1}H-NMR spectrum (700 MHz, D_2O) of dPG$_{500}$SA$_{0.20}$S$_{0.20}$.

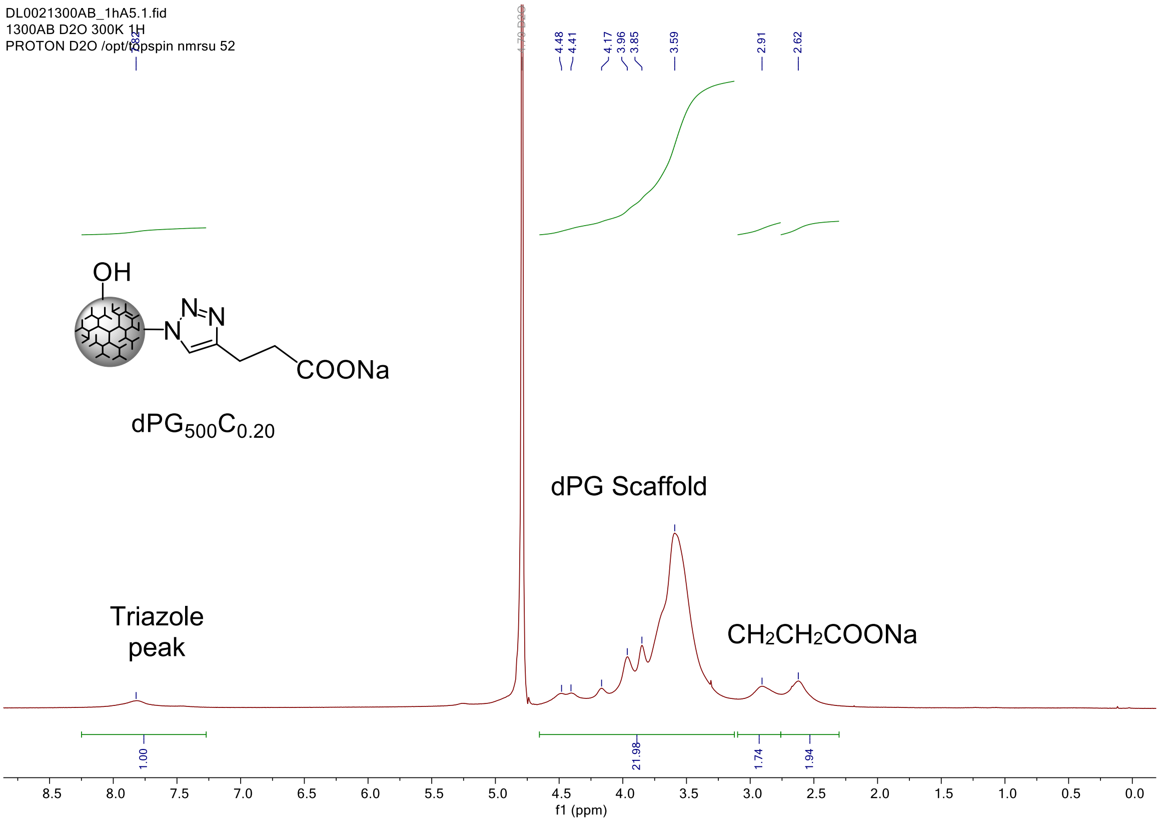

Figure S14: ^{1}H-NMR spectrum (500 MHz, D_2O) of $dPG_{500}C_{0.20}$.

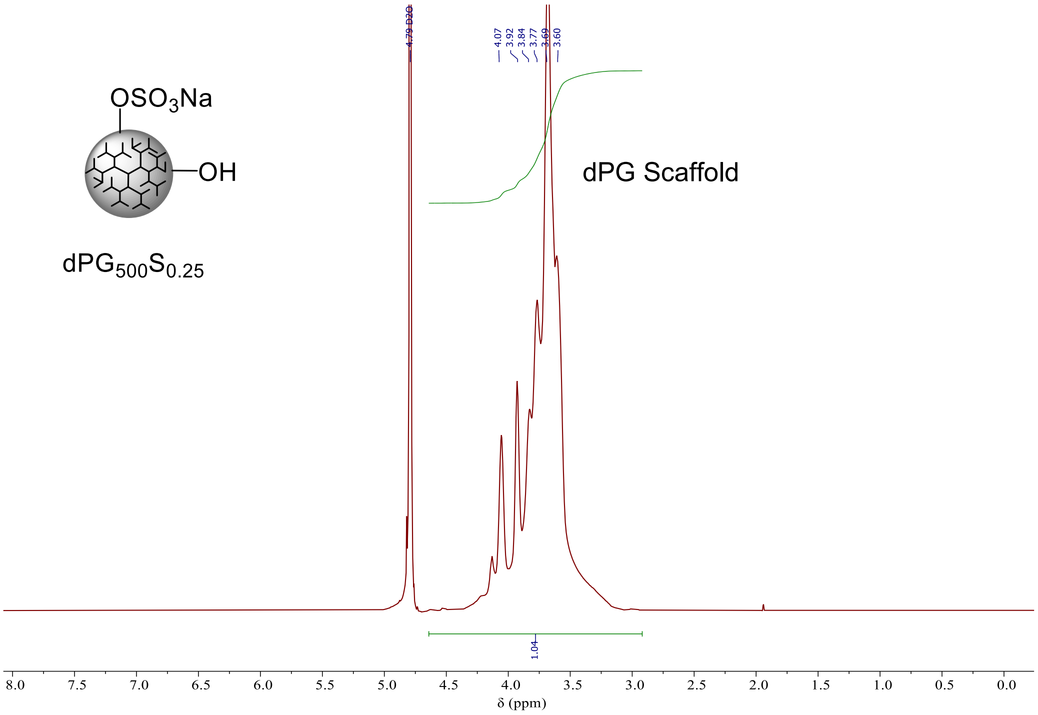

Figure S15: ^{1}H-NMR spectrum (700 MHz, D_2O) of $dPG_{500}S_{0.25}$.

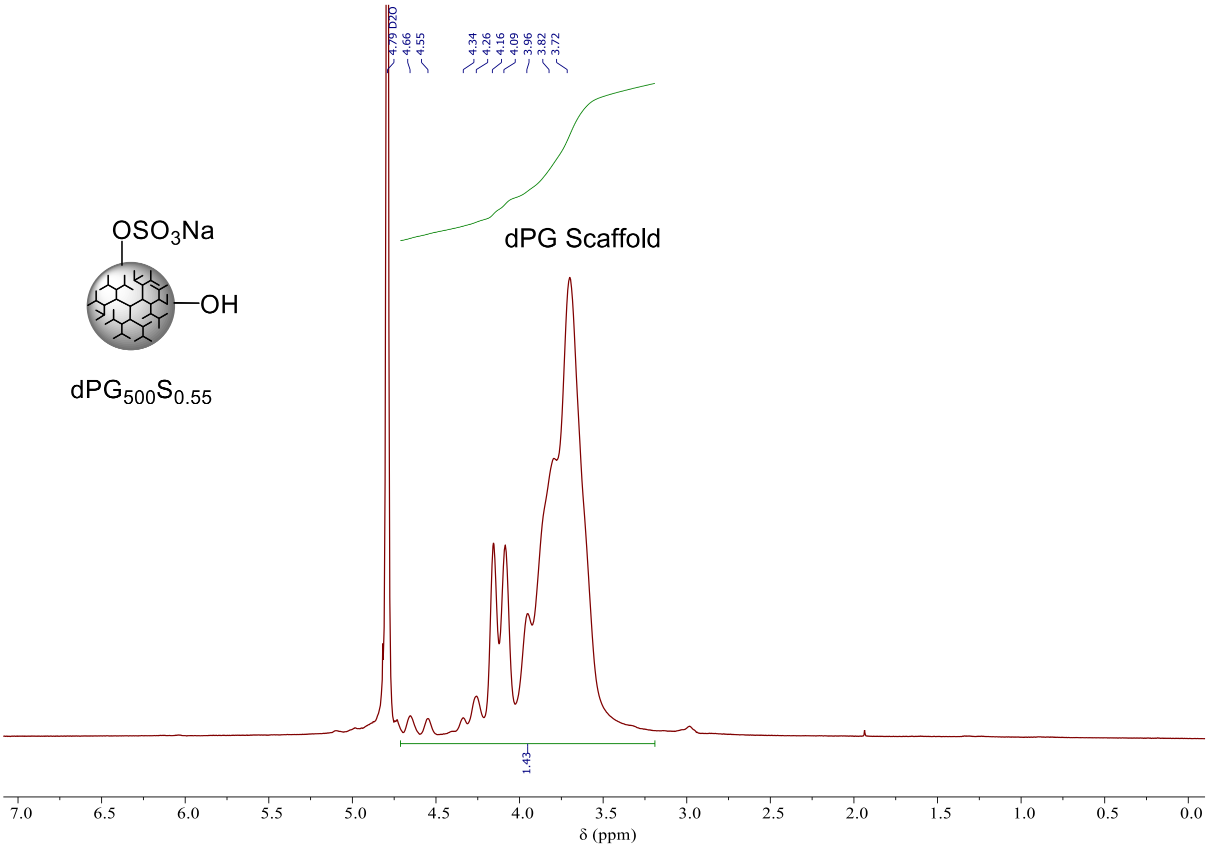

Figure S16: ^{1}H-NMR spectrum (700 MHz, D_2O) of $dPG_{500}S_{0.55}$.

2.2 DLS Plots

2.2 DLS Spectra

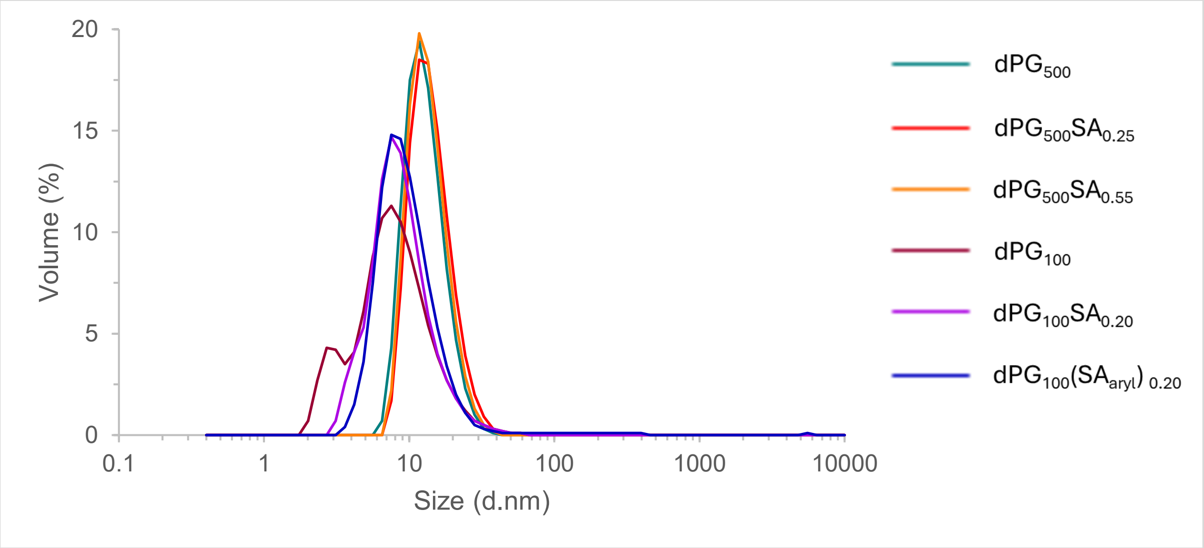

Figure S17: The volume distribution profile of 500 kDa and 100 kDa based dendritic polyglycerol and their sialoside conjugates, as observed by dynamic light scattering (DLS). All samples were analyzed at 1 mg/mL in aqueous PB (pH 7.4, 10 mM) at 25 ºC.

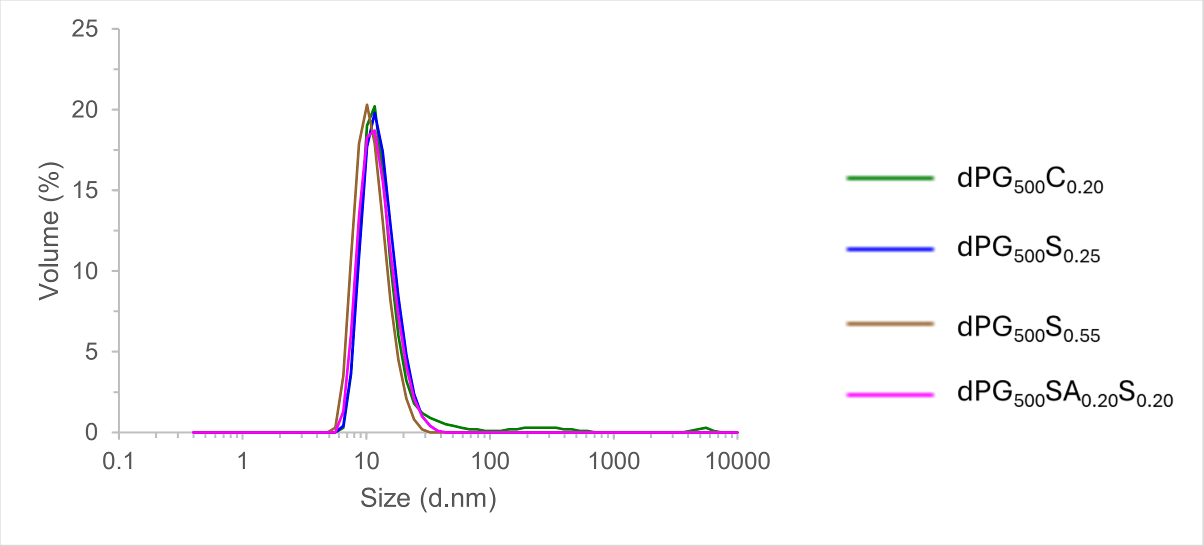

Figure S18: The volume distribution profile of 500 kDa based dendritic polyglycerolsulfates as observed by dynamic light scattering (DLS). All samples were analysed at 1 mg/mL in aqueous PB (pH 7.4, 10 mM) at 25 ºC.

2.3 Ensemble Docking Studies

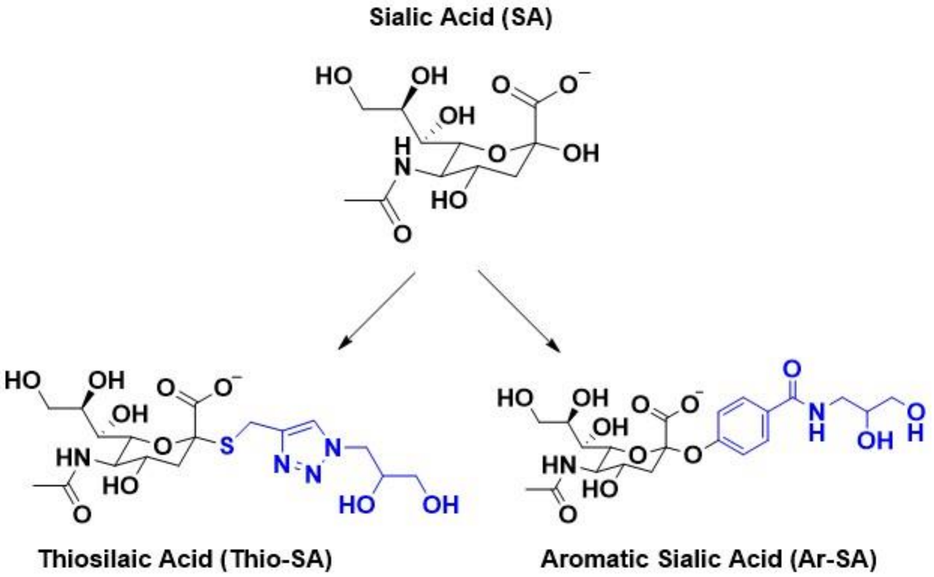

Scheme S2: Chemical structures of different sialic acid ligands taken for the ensemble docking studies: SA, Thio-SA and Ar-SA. Thio-SA and Ar-SA are both derivatives of SA. All three ligands have the same net charge of -1 e.

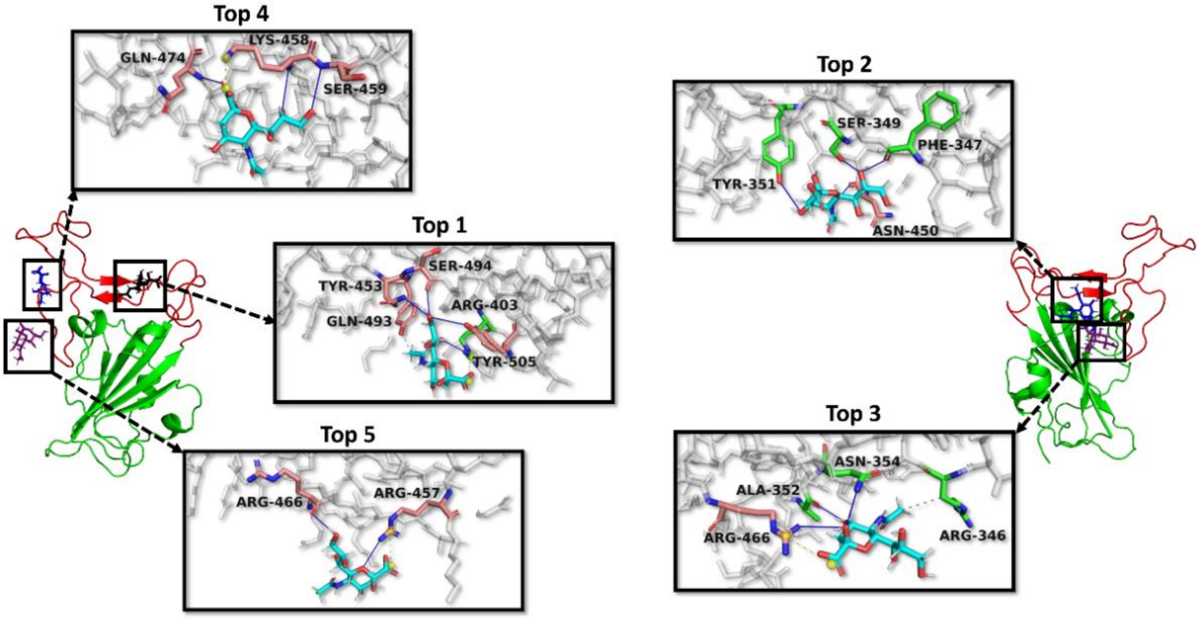

Figure S19: MD simulation snapshots highlighting ligand-protein interactions for the SA ligand. Carbons of ligand in cyan, carbons of ligand-bound RBD residues in green except carbons of RBM residues in salmon, oxygen in red, nitrogen in blue.

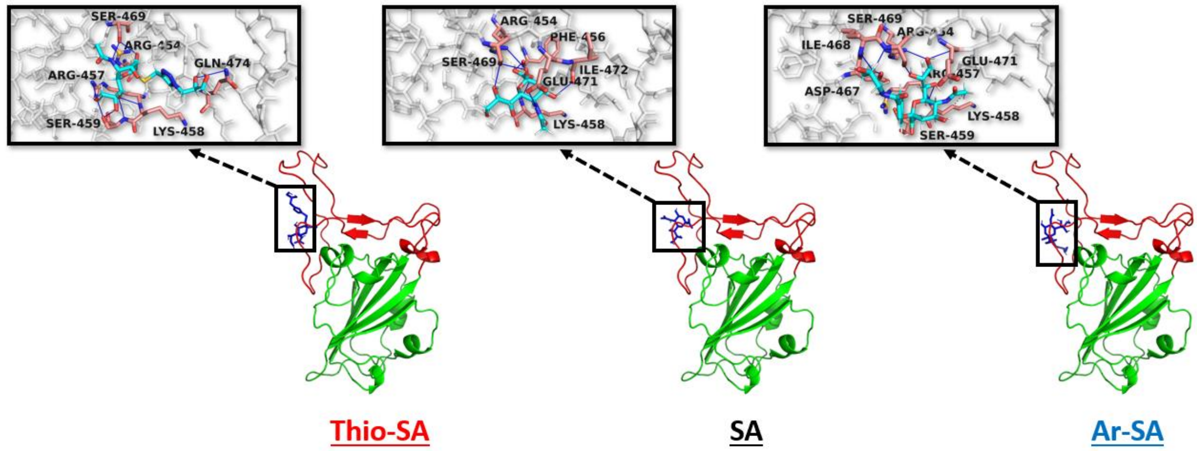

Figure S20: Snapshots highlighting ligand–protein interactions from the top docking poses for the three different ligands, SA, Thio-SA, and Ar-SA with the RBD. Carbons of ligand in cyan, carbons of ligand-bound RBD residues in green except carbons of RBM residues in salmon, oxygen in red, nitrogen in blue.

2.4 MD Simulation and Ensemble Docking Data Analysis

Number of close contacts: A close contact is counted if a ligand atom is within 3.5 Å of any atoms of a protein residue. The total number of such contacts is calculated for each protein residue and the average values are obtained from the last 900 ns of the simulation trajectory.

Number density: The number of ligands within a shell (of width 0.2 Å) at a distance *r* from the RBD surface is divided by the volume of the spherical shell to get the number density at that distance. The last 900 ns of the simulation trajectory is used to obtain the average number

density profile.

Residence time for different ligand functional groups: In order to examine the importance of different functional groups, specifically carboxylate (COO^-) and sulfate (SO_4^-), on the binding of sialic acid ligands to the RBD protein, we conduct the survival probability (SP) calculation using the autocorrelation module of the MDAnalysis package.[4,5] These functional groups are constituents of another ligand known as BGLC (part of the Heparin monomer), which is a derivative of β-D-Glucose. We use data from the MD simulation of RBD in a solution of BGLC ligands for the analysis. The timescale associated with the ligand binding–unbinding equilibrium can be determined from the SP analysis. We determine the normalized survival probability, $P(t)$, as[6]

$$P(t) = \left\langle \frac{N(t_0,\ t_0+t)}{N(t_0)} \right\rangle = \frac{1}{T}\sum_{t_0=1}^{T} \frac{N(t_0,\ t_0+t)}{N(t_0)},$$

where T is the total simulation time, $N(t_0)$ is the number of functional groups within the binding shell d at the initial time t_0, $N(t_0,\ t_0 + t)$ is the number of functional groups remaining in the binding shell d continuously from time t_0 to $t_0 + t$, the angular bracket represents the average over time origin t_0. This calculation provides the probability of a functional group remaining in the binding shell d for a specific time period t, indicating its continuous presence.

For the calculation, we use a window step of 10 ns (1000 frames) and set the maximum t value to 500 ns (50000 frames). SP for the different functional groups are shown in Figure S21. The normalized survival probability is fitted to a bi-exponential function:

$$P(t) = Ae^{-t/\tau_1} + Be^{-t/\tau_2} + C.$$

The decay time constants, τ_1 and τ_2, obtained from the fitting are also given in the inset of Figure S21 for both the functional groups, COO^- and SO_4^-. We define the residence time as the largest decay time τ_2.

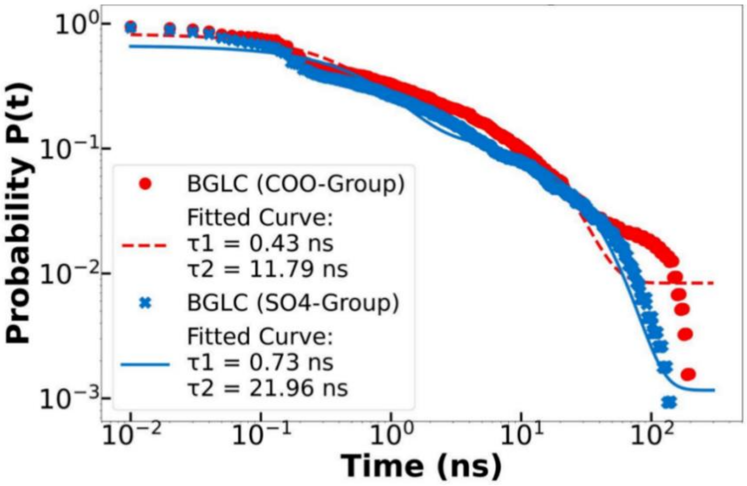

Figure S21: Survival probabilities of different functional groups (COO^- and SO_4^-) of the BGLC ligand. The decay time constants, τ_1 and τ_2, obtained from a biexponential fitting are provided. The residence time is defined as the largest decay time τ_2.

Binding free-energy difference from the residence time:

Consider a complexation reaction of protein p and ligand l to form protein–ligand pair pl, with the dissociation constant $K_\mathrm{d} = k_\mathrm{off}/k_\mathrm{on}$ given in terms of the on and off rates, k_on and k_off. The ligand binding free-energy ΔG_b (< 0) is given by

$$\Delta G_\mathrm{b} = k_\mathrm{B}T \ln(K_\mathrm{d}) = k_\mathrm{B}T \ln(k_\mathrm{off}/k_\mathrm{on}) = k_\mathrm{B}T \ln(1/k_\mathrm{on}\tau),$$

where k_B is the Boltzmann constant, T is the temperature, and $\tau = 1/k_\mathrm{off}$ is the residence time of the ligand in the bound state. For the same concentration of two different types of ligand, the on rate k_on depends on the diffusion constant, which can be obtained from MD simulations. Assuming k_on to be the same for the carboxylate and sulfate groups, since these two are part of the same ligand BGLC in our simulation, we obtain the binding free-energy difference between the two groups using their residence time values from Figure S21 as

$$\Delta\Delta G_\mathrm{b} = \Delta G_\mathrm{b}^{[\mathrm{SO}_4^-]} - \Delta G_\mathrm{b}^{[\mathrm{COO}^-]} = k_\mathrm{B}T \ln(\tau_{[\mathrm{COO}^-]}/\tau_{[\mathrm{SO}_4^-]}) = -0.62\ k_\mathrm{B}T.$$

Since both $\Delta G_\mathrm{b}^{[\mathrm{SO}_4^-]}$ and $\Delta G_\mathrm{b}^{[\mathrm{COO}^-]}$ are negative values, the sulfate group interacts with the SARS-CoV-2 RBD more strongly than the carboxylate group.

Ligand binding sites on the RBD: Recent studies have revealed that the receptor binding motif (RBM), consisting of the residues 438-506 of the RBD, has been identified as being directly involved in the interaction with the host cell receptor protein ACE2.[7] To identify the binding sites of sialic acid (SA) on the RBD, we conduct a comprehensive analysis of the protein residues interacting with SA found in the top binding poses from the ensemble docking

studies and in the five longest binding events obtained from the MD simulation involving SA ligands. Figure S22 depicts the time series of the SA ligand binding and unbinding events to the RBD. To ensure reliable results, we have discarded the first 100 ns MD simulation data for equilibration purpose and considered the last 900 ns of the MD simulation for the analysis. Table S1 provides a summary of the five longest binding events, including the residence time, time of occurrence, and corresponding ligand indices. Tables S2 and S3 comprise a comprehensive list of the binding residues obtained from the ensemble docking studies and the MD simulation, respectively. The residues belonging to RBM are highlighted in red in Tables S2 and S3.

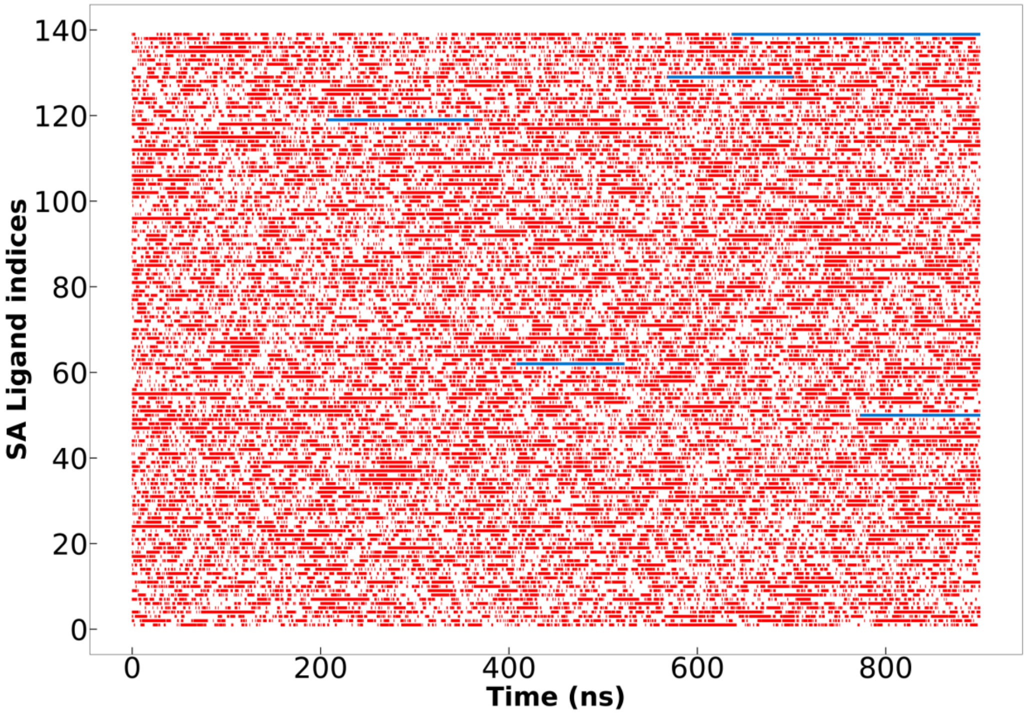

Figure S22: Time series of binding events of SA ligands to the RBD (white: unbound, red: bound, blue: five longest binding events).

Table S1: Data for the five longest SA ligand-binding events seen in the simulation.

Residence time (ns)	Time of binding events (ns)	Ligand indices
263.1	636.9 - 900.0	139
155.1	207.5 - 362.6	119
133.5	567.5 - 701.1	129
126.6	773.3 - 900.0	50
114.5	407.8 - 522.3	62

Table S2: Binding residues of the RBD from the top docking pose of SA, Thio-SA, Ar-SA ligands. Note that all binding residues belong to the receptor binding motif (RBM).

SA	Thio-SA	Ar-SA
454, 456, 458, 469, 471, 472	454, 457, 458, 459, 469, 474	454, 457, 458, 459, 467, 468, 469, 471

Table S3: Binding residues of the RBD selected from the five longest SA ligand-binding events seen in the simulation. Residues corresponding to the receptor binding motif (RBM) are colored red.

Binding event	Residence time (ns)	Binding residues
1.	263.1	403, 453, 493, 494, 505
2.	155.1	347, 349, 351, 450
3.	133.5	346, 352, 354, 466
4.	126.6	458, 459, 474
5.	114.5	457, 466

2.5 Cell Viability

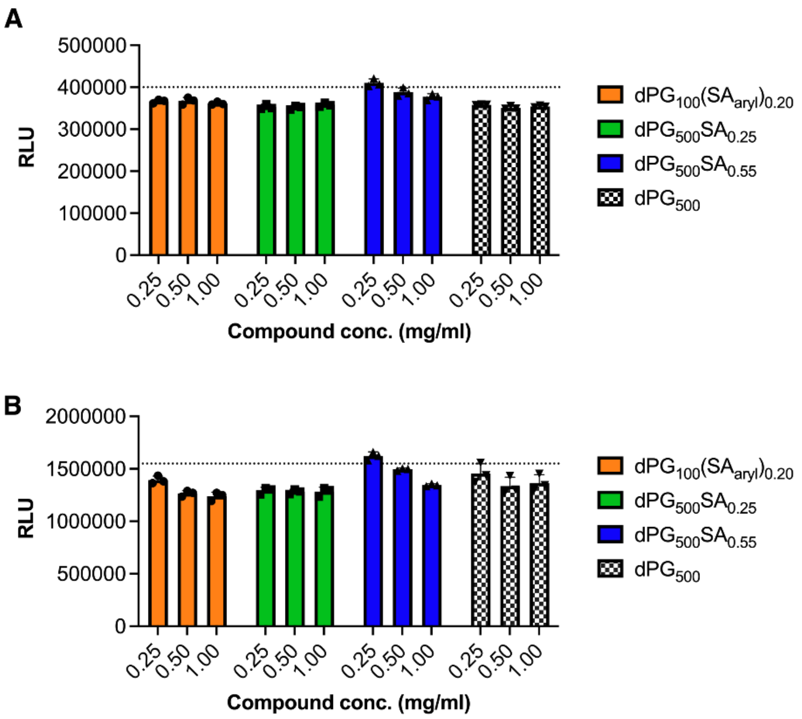

Figure S23: Cell viability of Calu-3 cells treated with the compounds.

(A – B) Calu-3 cells were treated with 0.25, 0.5 or 1 mg/ml of the indicated compounds. Cell viability was determined using CellTiter-Glo® Luminescent Cell Viability Assay at 24 hpi (A) and 48 hpi (B) from the supernatant of treated cells. Dotted lines represent mean values of

untreated cells. Data are represented as scatter plot with results from individual measurements from technical triplicate. The error bars represent standard deviation (SD).

2.6. XPS data

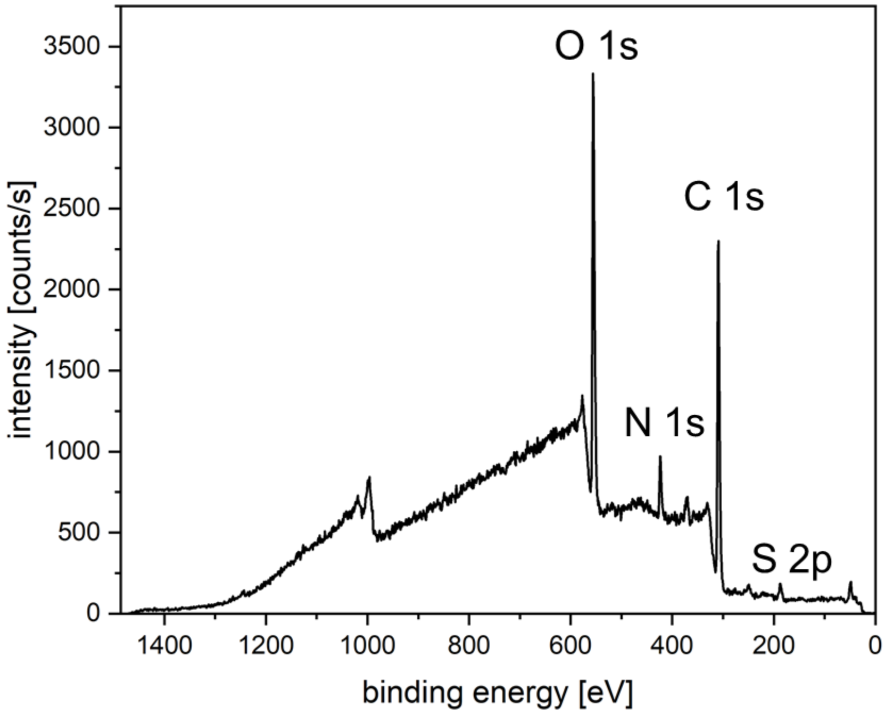

Figure S24: XPS spectrum of $dPG_{500}SA_{0.55}$. No peaks at binding energies around 933 eV and 952 eV are observed that confirm the absence of residual Cu ions in the sample.

References:

1. M. I. ul-haq, R. A. Shenoi, D. E. Brooks, J. N. Kizhakkedathu, Solvent-assisted anionic ring opening polymerization of glycidol: Toward medium and high molecular weight hyperbranched polyglycerols. *J. Polym. Sci. Part A: Polym. Chem.* **2013**, *51*, 2614.
2. S. Bhatia, D. Lauster, M. Bardua, K. Ludwig, S. Angioletti-Uberti, N. Popp, U. Hoffmann, F. Paulus, M. Budt, M. Stadtmüller, T. Wolff, A. Hamann, C. Böttcher, A. Herrmann, R. Haag, Linear polysialoside outperforms dendritic analogs for inhibition of influenza virus infection *in vitro* and *in vivo. Biomater.* **2017**, *138*, 22.

3. S. Roller, H. Zhou, R. Haag, High-loading polyglycerol supported reagents for Mitsunobu- and acylation-reactions and other useful polyglycerol derivatives. *Mol. Divers.* **2005**, *9*, 305.
4. N. Michaud-Agrawal, E. J. Denning, T. B. Woolf, O. Beckstein, MDAnalysis: A toolkit for the analysis of molecular dynamics simulations. *J. Comput. Chem.* **2011**, *32*, 2319.
5. R. J. Gowers, M. Linke, J. Barnoud, T. J. E. Reddy, M. N. Melo, S. L. Seyler, J. Domański, D. L. Dotson, S. Buchoux, I. M. Kenney, O. Beckstein, *Proc. of the 15th Python in Science Conf.* **2016**, pp. 98.
6. A. Debnath, B. Mukherjee, K. G. Ayappa, P. K. Maiti, S.-T. Lin, Entropy and dynamics of water in hydration layers of a bilayer. *J. Chem. Phys.* **2010**, *133*, 174704.
7. J. Lan, J. Ge, J. Yu, S. Shan, H. Zhou, S. Fan, Q. Zhang, X. Shi, Q. Wang, L. Zhang, X. Wang, Structure of the SARS-CoV-2 spike receptor-binding domain bound to the ACE2 receptor. *Nature* **2020**, *581*, 215.
